# Comparative effectiveness of antihypertensive monotherapies in primary prevention of cardiovascular events—a real-world longitudinal inception cohort study

**DOI:** 10.3389/fphar.2024.1357567

**Published:** 2024-06-05

**Authors:** Xuechun Li, Maarten J. Bijlsma, Stijn de Vos, Jens H. J. Bos, Sumaira Mubarik, Catharina C. M. Schuiling-Veninga, Eelko Hak

**Affiliations:** ^1^ PharmacoTherapy, Epidemiology and Economics, Groningen Research Institute of Pharmacy, University of Groningen, Groningen, Netherlands; ^2^ Max Planck Institute for Demographic Research, Rostock, Germany

**Keywords:** acute cardiac drug therapy, time-varying confounding, inverse probability weighting, Cox regression, comparative effectiveness

## Abstract

**Introduction:**

Antihypertensive drugs are used preventatively to lower the risk of cardiovascular disease events. Comparative effectiveness studies on angiotensin-converting enzyme inhibitors (ACEIs), angiotensin II receptor blockers (ARBs), beta-blockers (BBs), calcium channel blockers (CCBs), and thiazides have yielded inconsistent results and given little consideration to patient adherence. Using a longitudinal cohort and considering time-varying adherence and confounding factors, we aimed to estimate the real-world effectiveness of five major antihypertensive drug monotherapies in the primary prevention of cardiovascular events.

**Methods:**

Eligible patients for a retrospective inception cohort study were selected using information obtained from the University of Groningen IADB.nl pharmacy prescription database. Cohort 1 comprised adherent patients with a follow-up time exceeding 1 year, and cohort 2 comprised all patients independent of adherence. The exposures were ACEIs, ARBs, BBs, CCBs, and thiazides. The primary outcome was the time to the first prescription for an acute cardiac drug therapy (CDT) measured using valid drug proxies to identify the first major cardiovascular event. A per-protocol analytical approach was adopted with inverse probability of treatment weighted (IPTW), time-varying Cox regression analysis to obtain the hazard ratios (HRs) and 95% confidence intervals (CIs).

**Results:**

In cohort 1 (*n* = 22,441), 1,294 patients (5.8%) were prescribed an acute CDT with an average follow-up time of 4.2 ± 2.8 years. Following IPTW, the hazard measures of ARBs and thiazides were lower than those of BBs (HRs: 0.79 and 0.80, respectively; 95% CIs: 0.64–0.97 and 0.69–0.94, respectively). Among drug-treated diabetic patients, the hazard measures were even lower, with HR point estimates of 0.43 (CI: 0.19–0.98) for ARBs and 0.32 (CI: 0.13–0.82) for thiazides. In cohort 2 (*n* = 33,427) and sensitivity analysis, the comparative effectiveness results for thiazides and BBs were similar to those for cohort 1.

**Conclusion:**

The findings of this real-world analysis suggest that the incidence of CDT associated with long-term thiazide or ARB monotherapy is lower than the incidence of CDT with BBs, notably among high-risk patients. Incidences of CDT associated with ACEIs and CCBs were comparable relative to those associated with BBs.

## 1 Introduction

Antihypertensive drugs lower blood pressure and may be used preventatively to reduce the risk of cardiovascular disease (CVD) events (Black, 1998; Messerli et al., 2007). Angiotensin-converting enzyme inhibitors (ACEIs), angiotensin II receptor blockers (ARBs), beta-blockers (BBs), calcium channel blockers (CCBs), and thiazides are the five most common classes of drugs applied in hypertension treatment and CVD prevention worldwide (World Health Organization WHO, 2021). Some studies found that thiazide monotherapy performed better than BBs in the primary prevention of CVD (Psaty et al., 2003; Fretheim et al., 2012), while others found that the effectiveness of BBs was inferior or that there was no difference compared with the other four classes (Fretheim et al., 2012; Vögele et al., 2017; Suchard et al., 2019). Our previous intention-to-treat (ITT) analysis (Li et al., 2023), which had a 25-year follow-up time, also showed that individuals starting with thiazide treatment had a lower incidence of acute cardiac drug therapy (CDT) compared with those who started with BBs. In contrast, individuals starting with CCB monotherapy had a higher incidence of CDT compared with those starting with BBs. Incidences of CDT reported for ACEIs and ARBs were comparably relative to that for BBs. However, these studies did not consider confounding factors such as patient adherence or other time-varying risk factors for cardiovascular events.

Low adherence to drug prescriptions among hypertensive patients can influence the risk of CVD (Abegaz et al., 2017; Salazar, 2021). Previous studies on adherence have mostly focused on baseline or time-constant rates (Li et al., 2021), which could introduce bias in assessments of the associations between drug therapies and their outcomes. Because adherence can change over time, analyzing the comparative effectiveness of these five monotherapies while considering time-varying variables can yield more accurate results.

Importantly, there is a paucity of studies on comparative effectiveness within different subgroups delineated by sex, age, or common high-risk comorbidities. The World Health Organization (WHO) recommends the use of ACEIs/ARBs for diabetic patients (WHO, 2021), whereas no such recommendation is applicable in the case of rheumatoid arthritis (RA) or asthma/chronic obstructive pulmonary disease (COPD). The current guidelines (Genootschap and en Innovatie, 2019; Visseren et al., 2021; WHO, 2021) do not recommend a specific monotherapy, which makes these drugs appropriate objects of study with lower chances of confounding by indication. In this study, we estimated the relative effectiveness of five major classes of antihypertensive drug monotherapy in preventing cardiovascular events while considering time-varying confounders, such as adherence and use of co-medications, over a 10-year follow-up period.

## 2 Material and methods

### 2.1 Study design

We designed a retrospective inception cohort study, entailing an analysis of pre-existing data collected from individuals with a shared starting point or index date, who were then followed over a maximum period of 10 years during the study period between 1 January 1996 and 31 December 2020. The data were extracted from the IADB.nl prescription database maintained by the University of Groningen (Visser et al., 2013), which contains patient information similar to that used in our previous study (Li et al., 2023). IADB.nl includes prescriptions and other information gathered from 54 community pharmacies located in different parts of the Netherlands from 1994 to date. Diagnoses are not linked to the prescription data. The data contain each patient’s personal identification code (pseudonymized), date of birth, and sex. Prescription information includes the date of prescriptions and the associated Anatomical Therapeutic Chemical (ATC) code.

### 2.2 Study population

#### 2.2.1 Inclusion and exclusion criteria

**FIGURE 1:**
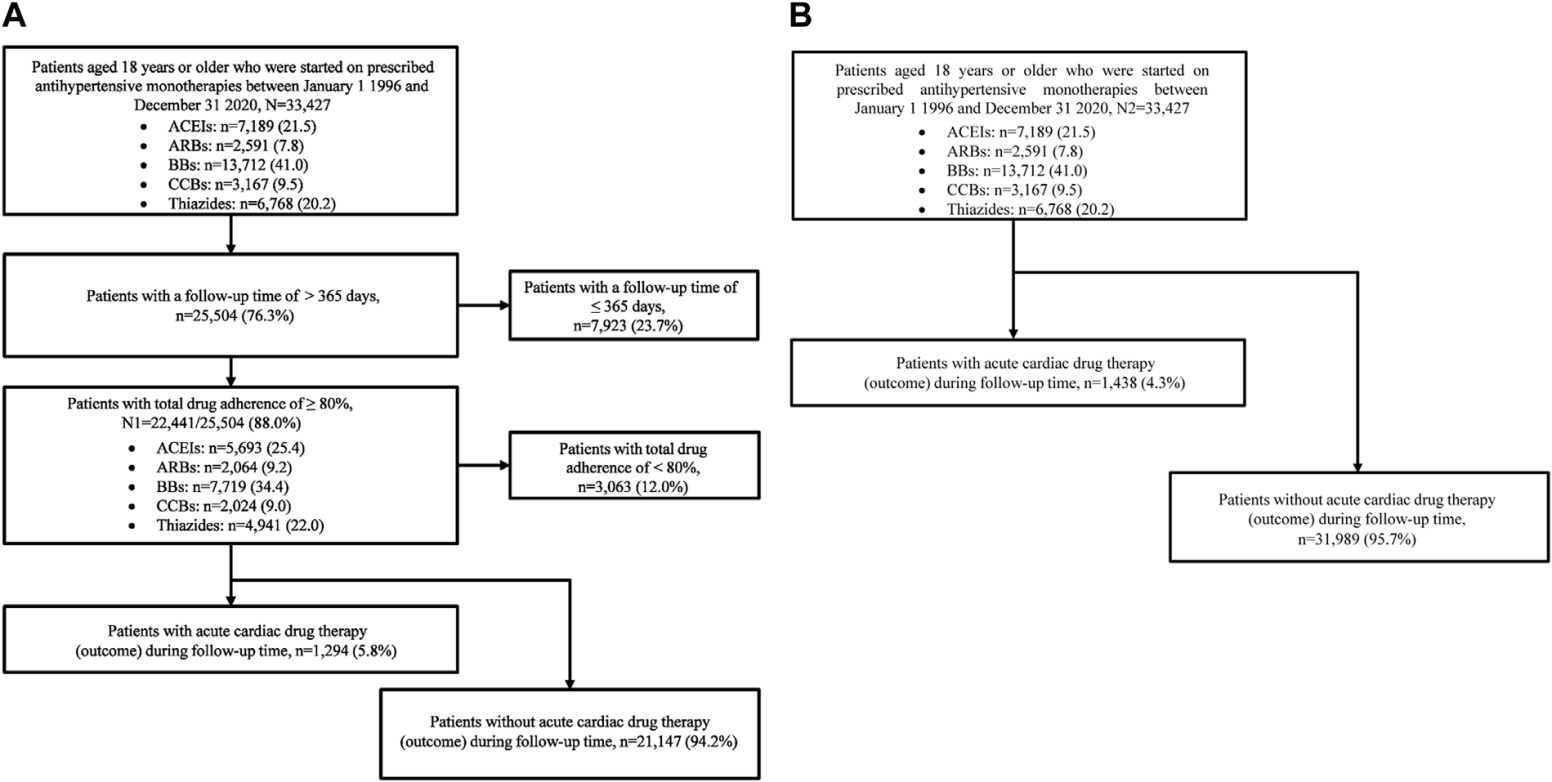
Flowcharts in cohort 1 and cohort 2 (N1 = 22,441 patients; N2 = 33,427 patients). **(A)** Cohort 1 flowchart. **(B)** Cohort 2 flowchart. Abbreviations: ACEIs, angiotensin-converting enzyme inhibitors; ARBs, angiotensin II receptor blockers; BBs, beta-blockers; CCBs, calcium channel blockers.

Patients were eligible for inclusion if they were aged 18 years or older on the index date. In this study, we divided our population into two groups according to total adherence (adherence_total_) and follow-up time (see below). Cohort 1 comprised patients who demonstrated high levels of adherence_total_ (adherence_total_ ≥80%), with a follow-up time that exceeded 1 year (>365 days) (Figure 1A), and cohort 2 included all patients, independent of their adherence_total_ (Figure 1B).

Furthermore, each patient had at least 2 years of prescription records prior to the index date and at least 1 year of records after this date. Every included patient had at least three prescriptions of the same class of antihypertensive monotherapy in the year commencing from the index date.

Patients who received antihyperlipidemic monotherapies within a year of the index date were excluded. This is because our primary objective was to determine the real-world effectiveness of monotherapy with antihypertensive treatment alone. As antihyperlipidemic agents can also reduce the occurrence of cardiovascular events, we excluded such patients to eliminate any bias. We also excluded patients who had received a prescription of more than two classes of antihypertensive drugs as monotherapies during the first year after the index date, with each type of monotherapy including at least three of the same classes of prescriptions at the third or fourth level of the ATC code (see the definition of exposure). We excluded patients who were taking at least two prescribed, fixed-dose antihypertensive or antihyperlipidemic drug combinations during the year that followed the index date. The reason we did not analyze the combination drugs is that most patients are first treated with monotherapies in the Netherlands, and only a few are given combination drugs at the outset. Therefore, our study exclusively focused on the effects of monotherapies. We also excluded patients who were treated with any acute CDT, as discussed below, within 2 years prior to the index date or within 90 days after this date. Patients on chronic drug therapy for stable heart failure (Whocc, 2020), migraine (Alfian et al., 2019), adrenal disease, hyperparathyroidism, and thyroid problems (at least two prescriptions) during the 2 years before the index date or within 90 days after it were also excluded (for the ATC codes of the treatment, see Supplementary Table S1).

#### 2.2.2 Follow-up

**FIGURE 2:**
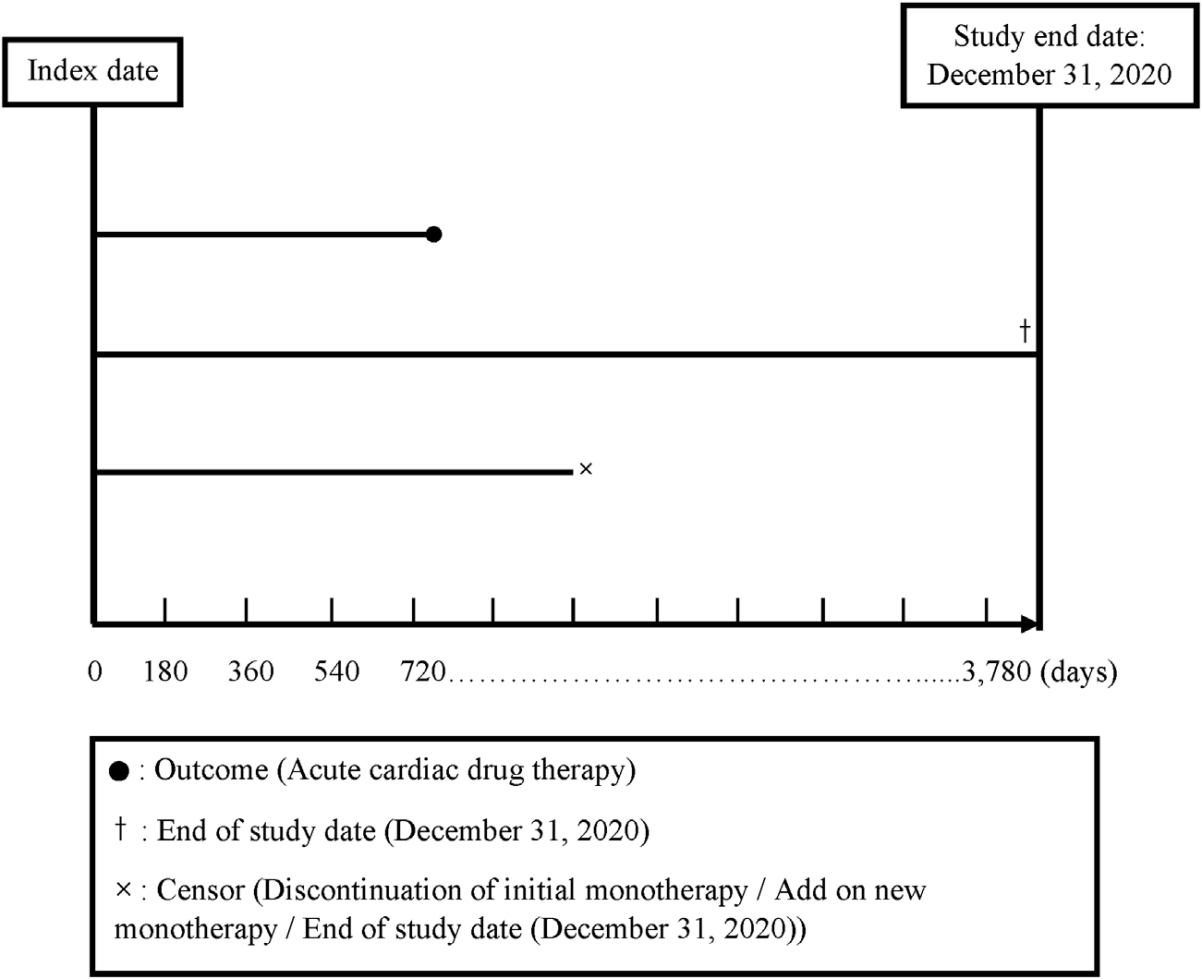
Survival times and censoring times in days.

We divided the total follow-up time into 180-day intervals, starting from the index date and continuing up to the end of follow-up (a maximum of 10 years: 3,780 days). The index date was defined as the date of the initial prescription of any antihypertensive monotherapy (see further content). Discontinuation was defined as not receiving a prescription of the same class for any of the five classes of antihypertensive drug monotherapy for more than 180 days (Corrao et al., 2011; Shau et al., 2019). A drug switch was defined as a patient’s receipt of a prescription for a new class of antihypertensive drug monotherapy or antihypertensive drug fixed-dose combination within 180 days following discontinuation of a specific drug therapy (a gap >180 days; Alfian et al., 2019). Drug add-on was defined as a new drug class prescribed in addition to an existing drug class prior to discontinuation (Nishimura et al., 2019) (for the follow-up plot, see Figure 2).

The end of follow-up was defined as (1) the date when the patients received the first prescription for a drug indicating an acute CDT; (2) the date when the patients had no outcome but discontinued or received an add-on antihypertensive drug to avoid any bias due to increased effects of the additional drug; or (3) the end of the 10-year follow-up period or 31 December 2020, whichever came first at the end of the follow-up time (Figure 2). Because the drug switch always occurred after the patients’ discontinuation of a treatment, it did not influence the ending of the follow-up time.

In our study, we applied two versions of adherence (Bijlsma et al., 2016; Shau et al., 2019). One version was time-constant (Adherence_total_; see Formula 1), and the other version was time-varying (Adherence_180_; see Formula 2). Both measures of adherence yielded proportions ranging from 0 to 1 (Corrao et al., 2011).

$$\text{Adherenc}e_{\text{total}}=\frac{\text{the}\;\text{total}\;\text{number}\;\text{of}\;\text{days}\;\text{covered}\;\text{by}\;\text{the}\;\text{antihypertensive}\;\text{drugs}\;\text{monotherapy}}{\text{the}\;\text{total}\;\text{number}\;\text{of}\;\text{follow}‐\text{up}\;\text{days}}.$$ (1)

$$\text{Adherenc}e_{180}=\frac{\text{the}\;\text{number}\;\text{of}\;\text{days}\;\text{covered}\;\text{in}\;a\;\text{given}\;180‐\text{day}\;\text{interval}}{\text{the}\;\text{number}\;\text{of}\;\text{follow}‐\text{up}\;\text{days}\;\text{in}\;a\;\text{given}\;180‐\text{day}\;\text{interval}}.$$ (2)

#### 2.2.3 Exposure

We considered the following five common antihypertensive monotherapies as the exposures in our study: thiazides (ATC code: C03AA), CCBs (ATC codes: C08C, C08D, and C08E), ACEIs (ATC code: C09A), ARBs (ATC code: C09C), and BBs (ATC code: C07A). Different chemical compounds within one specific monotherapy group (those with the same ATC code at levels 3 or 4) were deemed to belong to the same class of antihypertensive drug monotherapy.

#### 2.2.4 Primary outcome

Our primary outcome was the time to the initial prescription of an acute CDT. Following Pouwels et al. (2016), we used acute CDT as a proxy for a major cardiovascular event. New CVD events were signified by the commencement of any of the following acute CDT drugs, given the high level of specificity (94%) required for causal studies. These drugs included a platelet aggregation inhibitor (B01AC), an organic nitrate (C01DA), and/or a vitamin K antagonist (B01AA) or other vasodilators used in acute CDT (C01DX), with at least two prescriptions of any of these drugs during a 180-day period following the index date.

#### 2.2.5 Potential confounders

The individual’s sex and age on the index date were recorded (Table 1). The initial drug therapy at the baseline for diabetes, RA, or asthma/COPD was defined as at least one prescription within the first 180-day period following the index date. A calendar year was defined as the year of the initial prescription of an exposure drug. Time-varying drug therapies for diabetes/RA/asthma/COPD during the follow-up time were defined according to whether a prescription was in effect during any of the 180-day periods following the index date.

**TABLE 1:** Variables related in cohort 1 and cohort 2.

	Time constant^a^	Time varying^b^	IPTW adjustment^c^	Subgroup analysis^d^
Cohort 1 (Adherence_total_ ≥80% with at least 1 year follow-up time)	• Sex, age(continuous), age(category), calendar years, and adherence_**total**_	• Drugs for diabetes/RA/asthma or COPD during the follow-up time	• Sex, age (continuous), and calendar years	• Sex, age (category), and calendar years
	• Initial drugs used for diabetes/RA/asthma or COPD		• Drugs for diabetes/RA/asthma or COPD	• Initial drugs used for diabetes/RA/asthma or COPD
Cohort 2 (all patients independent of adherence)	• Sex, age (continuous), age(category), calendar years, and adherence_total_	• Drugs for diabetes/RA/asthma or COPD during the follow-up time	• Sex, age (continuous), calendar years, and adherence_180_	• Sex, age (category), calendar years, and adherence_total_
	• Initial drugs used for diabetes/RA/asthma or COPD	• adherence_180_	• Drugs for diabetes/RA/asthma or COPD	• Initial drugs used for diabetes/RA/asthma or COPD

Notes:

^a^ All related time-constant variables in two cohorts.

^b^ All related time-varying variables in two cohorts.

^c^ Variables used for IPTW adjustment with exposure.

^d^ Variables used as different subgroup analysis.

Abbreviations: IPTW, inverse probability treatment weighting; RA, rheumatoid arthritis; COPD, chronic obstructive pulmonary disease.

### 2.3 Statistical analysis

The R package was used for data preprocessing and analysis. Baseline characteristics were summarized as mean ± standard deviations and frequencies for continuous and categorical variables, respectively. We used Pearson’s χ2 test and Welch’s ANOVA test function in R to test for differences in the baseline characteristics between the groups, and we set α at 0.05, indicating statistical significance for the two-sided test.

We used the survfit function in the “survival” package to plot Kaplan–Meier survival curves of time to acute CDT for the four different classes of drugs compared with BBs, with and without inverse probability treatment weighting (IPTW) for patients in cohorts 1 and 2. This is because IPTW can correct for time-varying confounders and fit of a model for the regression of outcomes of interest and exposures of interest using observational data. We used the ipwtm function in the “ipw” R package (Van der Wal and Geskus, 2011) to calculate the stabilized propensity scores and fit the marginal structural models, with time-varying confounders in cohorts 1 and 2, respectively (Table 1, models 1 and 2).

Exposure=four types of antihypertensive monotherapy and BBs,

numerator=∼ sex+age+calendar year,

denominator=∼ sex+age+calendar year+drugs for diabetes+drugs for RA+drugs for asthma or COPD.

Model 1

*Time-constant variables: sex, age, and calendar year.
**Time-varying confounders: drugs for diabetes, RA, and asthma or COPD.

Exposure=four types of antihypertensive monotherapy and BBs,

numerator=∼ sex+age+calendar year,

$$\text{denominator}=∼\text{ sex}+\text{age}+\text{calendar year}+\text{drugs for diabetes}+\text{drugs for RA}+\text{drugs for asthma or COPD}+{\text{adherence}}_{180}.$$

Model 2

*Time-constant variables: sex, age, and calendar year.
**Time-varying confounders: drugs for diabetes, RA, and asthma or COPD, adherence_180_.

We used the coxph function in the survival R package to construct a time-varying Cox regression model (Therneau et al., 2017) with and without IPTW. Consequently, we estimated the total relative effectiveness and relative effectiveness within the subgroups of the five antihypertensive drug monotherapies. We performed subgroup analysis for each category of time-constant variable, namely, sex, age, initial drugs used for diabetes/RA/asthma/COPD, the calendar year when treatment with the exposure drug commenced, and adherence_total_ (Table 1). For each of these subgroups, we repeated Cox regression with and without IPTW to calculate the crude hazards and IPTW-adjusted hazards. For the IPTW-adjusted Cox regression, we used the original propensity score generated from the overall IPTW adjustment performed for cohorts 1 and 2. We also estimated the effect of the post-IPTW interaction between each subgroup variable and our exposure. All the R codes are available from the authors upon request.

### 2.4 Sensitivity analysis

To assess the robustness of our results, we conducted sensitivity analyses for patients whose drugs were not switched, those who did not have any additional drug prescriptions, or those with only drug switches or drug additions during the 10-year and 5-year follow-up times, respectively (Table 2).

**TABLE 2:** Cohorts for sensitivity analysis.

Cohort name	Target population
Cohort 3	• Patients without drug switching and without drug add-on in adherent patients in 10-year follow-up time
	• Patients without drug switching and without drug add-on in adherent patients in 5-year follow-up time
Cohort 4	• Patients without drug switching and without drug add-on in all the patients in 10-year follow-up time
	• Patients without drug switching and without drug add-on in all the patients in 5-year follow-up time
Cohort 5	• Only patients with drug switching or drug add-on in adherent patients in 10-year follow-up time
	• Only patients with drug switching or drug add-on in adherent patients in 5-year follow-up time
Cohort 6	• Only patients with drug switching or drug add-on in all the patients in 10-year follow-up time
	• Only patients with drug switching or drug add-on in all the patients in 5-year follow-up time

## 3 Results

### 3.1 Baseline characteristics

The patients in cohort 1 had an average follow-up time of 4.2 ± 2.8 years (Table 3), whereas those in cohort 2 had a follow-up time of 3.2 ± 2.9 years (Supplementary Table S2). Of the 33,427 patients in cohort 2, fewer than 25% were followed for less than a year (≤365 days) because of the extended gap (>180 days) between the antihypertensive drug monotherapy prescriptions (Figure 1A).

**TABLE 3:** Baseline characteristics for cohort 1 population who used antihypertensive drug monotherapy.

Demographics	Total	ACEIs	ARBs	BBs	CCBs	Thiazides	*P* ^b^
	N = 22,441	N = 5,693 (25.4)^a^	N = 2,064 (9.2)^a^	N = 7,719 (34.4)^a^	N = 2,024 (9.0)^a^	N = 4,941 (22.0)^a^	
	n (%)	n (%)	n (%)	n (%)	n (%)	n (%)	
Average follow-up years^c^	4.2 ± 2.8	4.1 ± 2.8	4.3 ± 2.9	4.4 ± 3.0	3.6 ± 2.4	4.0 ± 2.8	—
Sex							<0.001
Male	10,036 (44.7)	3,259 (57.2)	1,039 (50.3)	2,934 (38.0)	963 (47.6)	1,841 (37.3)	
Age at entry^d^ (years)	57.7 ± 13.7	57.4 ± 13.5	57.7 ± 12.9	54.7 ± 14.2	60.0 ± 13.0	61.8 ± 12.6	<0.001^e^
18–39	2,054 (9.2)	504 (8.9)	154 (7.5)	1,099 (14.2)	124 (6.1)	173 (3.5)	<0.001
40–69	15,681 (69.9)	4,038 (70.9)	1,500 (72.7)	5,373 (69.6)	1,422 (70.3)	3,348 (67.8)	
≥70	4,706 (21.0)	1,151 (20.2)	410 (19.9)	1,247 (16.2)	478 (23.6)	1,420 (28.7)	
Initial comorbidity drug use							
Diabetes drug: yes	1,359 (6.1)	829 (14.6)	151 (7.3)	150 (1.9)	58 (2.9)	171 (3.5)	<0.001
RA drug: yes	219 (1.0)	64 (1.1)	23 (1.1)	51 (0.7)	40 (2.0)	41 (0.8)	<0.001
Asthma/COPD drug: yes	1,790 (8.0)	480 (8.4)	192 (9.3)	452 (5.9)	202 (10.0)	464 (9.4)	<0.001
Calendar-year periods							<0.001
1996–2000	1,523 (6.8)	356 (6.3)	91 (4.4)	712 (9.2)	98 (4.8)	266 (5.4)	
2000–2010	9,230 (41.1)	1,942 (34.1)	822 (39.8)	3,831 (49.6)	397 (19.6)	2,238 (45.3)	
2010–2020	11,688 (52.1)	3,395 (59.6)	1,151 (55.8)	3,176 (41.1)	1,529 (75.5)	2,437 (49.3)	

Notes:

^a^ Row percentage; others are all column percentage.

^b^ *p* value: significance value of the chi-squared test or ANOVA test, which showed the difference of the distribution of patients who used five antihypertensive monotherapies at the baseline in different subgroups of covariates.

^c^ Use mean ± standard deviations to describe average follow-up years.

^d^ Use mean ± standard deviations to describe continuous age.

^e^ Welch’s ANOVA test to describe whether patients of different classes of antihypertensive monotherapy were different in age (heterogeneity of variance).

Abbreviations: ACEIs, angiotensin-converting enzyme inhibitors; ARBs, angiotensin II receptor blockers; BBs, beta-blockers; CCBs, calcium channel blockers; RA, rheumatoid arthritis; COPD, chronic obstructive pulmonary disease.

### 3.2 Survival analysis

**FIGURE 3:**
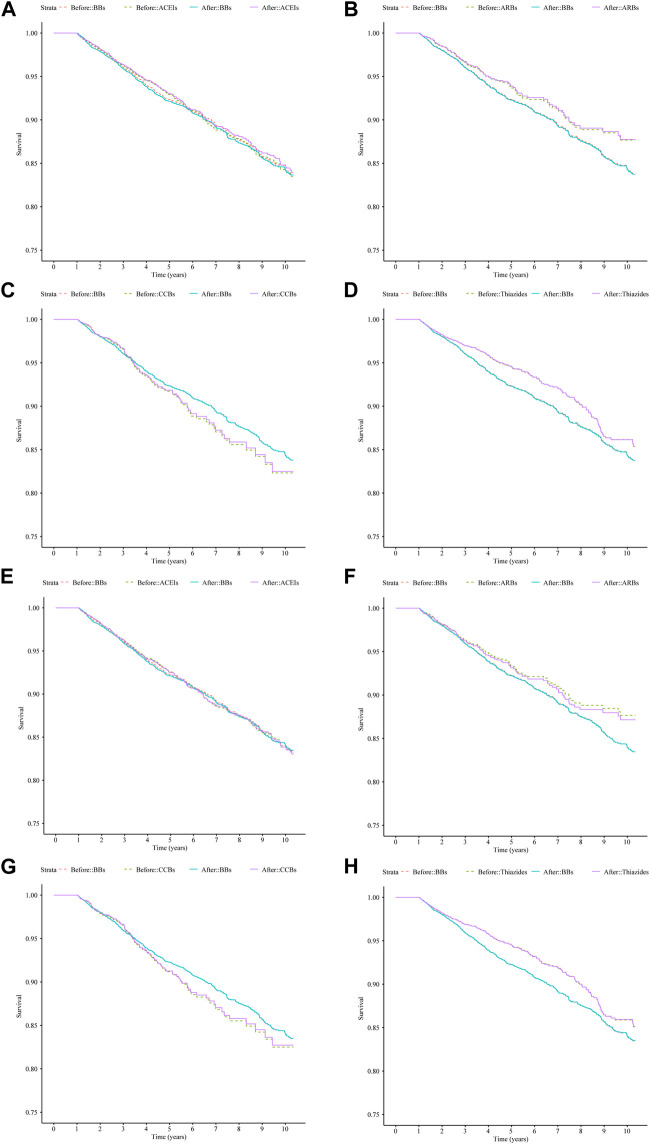
Survival curves for acute CDT in cohort 1 and cohort 2 patients treated with four types of antihypertensive monotherapies compared with BBs in 10 years before and after IPTW; **(A–D)**: cohort 1 patients; **(E–H)**: cohort 2 patients. **(A)** ACEIs vs. BBs (cohort 1), **(B)** ARBs vs. BBs (cohort 1), **(C)** CCBs vs. BBs (cohort 1), **(D)** thiazides vs. BBs (cohort 1), **(E)** ACEIs vs. BBs (cohort 2), **(F)** ARBs vs. BBs (cohort 2), **(G)** CCBs vs. BBs (cohort 2), and **(H)** thiazides vs. BBs (cohort 2). Notes: before: time-varying Cox regression before IPTW adjustment; after: time-varying Cox regression after IPTW adjustment. Abbreviations: IPTW, inverse probability treatment weighting; CDT, cardiac drug therapy; ACEIs, angiotensin-converting enzyme inhibitors; ARBs, angiotensin II receptor blockers; BBs, beta-blockers; CCBs, calcium channel blockers.

After IPTW adjustment, there were minimal differences between the survival curves for the four classes of monotherapy and the BBs compared with the curves prior to IPTW adjustment for both cohorts (Figure 3). ACEIs and BBs evidenced similar trends. ARBs and thiazides had a higher survival rate, while CCBs had a lower survival rate. The hazard measures of acute CDT were lower for thiazides than for the reference BBs in both cohorts 1 and 2 before and after IPTW adjustment, whereas they were lower for ARBs only in cohort 1 (Table 4).

**TABLE 4:** Cox regression analysis of acute CDT in cohort 1 and cohort 2

	Acute CDT in cohort 1				Acute CDT in cohort 2			
Antihypertensive monotherapies	Crude HR^a^ (95%CI)	*p*	IPTW adjusted^b^ HR (95% CI)	*p*	Crude HR^c^ (95% CI)	*p*	IPTW adjusted^d^ HR (95% CI)	*p*
Reference: BBs								
Exposure								
ACEIs	0.99 (0.86.1.14)	0.9	0.93 (0.80.1.08)	0.358	1.00 (0.88.1.14)	0.997	0.98 (0.85.1.14)	0.804
ARBs	0.81 (0.66.1.00)	0.047	0.79 (0.64.0.97)	0.027	0.82 (0.68.1.00)	0.053	0.85 (0.69.1.05)	0.136
CCBs	1.10 (0.90.1.36)	0.344	1.08 (0.88.1.33)	0.482	1.10 (0.91.1.34)	0.327	1.08 (0.89.1.32)	0.439
Thiazides	0.81 (0.69.0.95)	0.008	0.80 (0.69.0.94)	0.005	0.80 (0.69.0.93)	0.004	0.80 (0.69.0.93)	0.004

Notes:

^a^ Crude Cox regression model only containing antihypertensive monotherapies and CDT outcome.

^b^ IPTW adjusted between antihypertensive monotherapies and sex, age, drugs for diabetes, drugs for RA, drugs for asthma/COPD, and calendar-year periods.

^c^ Crude Cox regression model only contains antihypertensive monotherapies and CDT outcome.

^d^ IPTW adjusted between antihypertensive monotherapies and sex, age, drugs for diabetes, drugs for RA, drugs for asthma/COPD, calendar-year periods, and adherence_180_ in each period.

Abbreviations: CDT, cardiac drug therapy; HR, hazard ratio; CI, confidence interval; IPTW, inverse probability treatment weighting; ACEIs, angiotensin-converting enzyme inhibitors; ARBs, angiotensin II receptor blockers; BBs, beta-blockers; CCBs, calcium channel blockers.

### 3.3 Subgroup and interaction analysis

**FIGURE 4:**
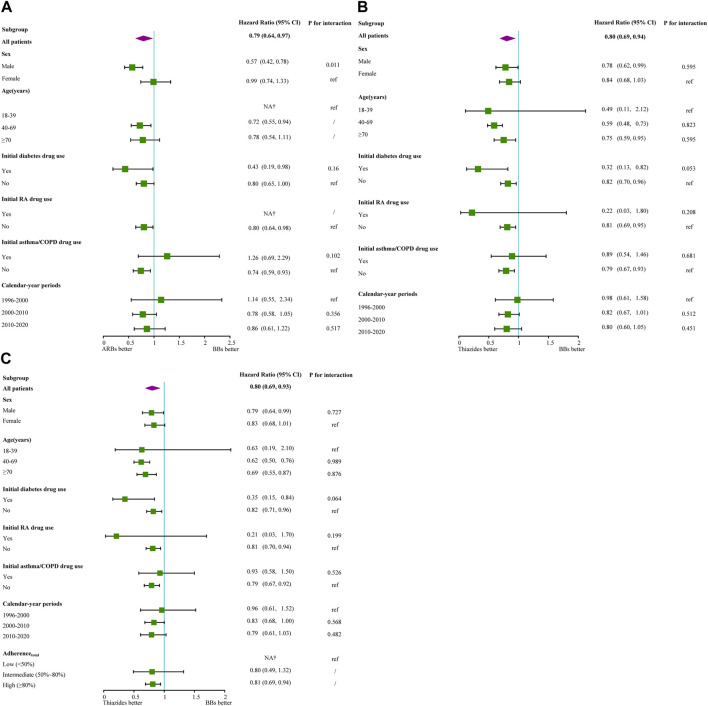
Forest plots of subgroup hazard ratios between ARBs compared with BBs in cohort 1 after IPTW and thiazides compared with BBs after IPTW in cohort 1 and cohort 2. **(A)** ARBs vs. BBs in cohort 1, **(B)** thiazides vs. BBs in cohort 1, **(C)** thiazides vs. BBs in cohort 2. Notes: *p* for interaction: Cox regression model contain treatment, confounding variables, and their interaction term. ref: reference group.†: number of events too small for effect size calculation. /: If the HR of the reference group or control group was NA, then we did not consider the *p* for interaction of the control group. Abbreviations: CI, confidence interval; ARBs, angiotensin II receptor blockers; BBs, beta-blockers; RA, rheumatoid arthritis; COPD, chronic obstructive pulmonary disease; IPTW, inverse probability treatment weighting.

Following IPTW, consistent results were obtained for the comparison of ARBs and BBs in cohort 1 and 2 and between thiazides and BBs in some subgroups in this study (Supplementary Tables S3, S4). Although point estimates indicated higher relative effectiveness of ARBs and thiazides compared with BBs in the risk groups, the interaction estimates were not significant for ARBs and thiazides compared with those for BBs. However, the sex of the patients in cohorts 1 and 2 showed an interaction effect in the comparison between ARBs and BBs (Figure 4; Supplementary Tables S3, S4).

### 3.4 Sensitivity analysis

The 5-year and 10-year Kaplan–Meier curves for the patients in cohorts 3 and 4 showed almost the same trends as those obtained for cohorts 1 and 2 (Supplementary Figure S1). This similarity between the two groups of cohorts was also observed in the Cox regression analysis (Supplementary Table S5).

In cohorts 5 and 6, the Kaplan–Meier curves of ARBs and BBs, thiazides and BBs, and CCBs and BBs showed the same 5-year and 10-year trends found for cohorts 1 and 2. ACEIs and CCBs were associated with lower survival rates than BBs during the 10-year follow-up period. However, for the 5-year follow-up period, the survival rate was higher for ACEIs than for BBs (Supplementary Figure S2). The similarity of the Kaplan–Meier curves was also observed in the Cox regression analysis results (Supplementary Table S6).

## 4 Discussion

We found that the incidence of CDT was lower with thiazide monotherapy compared with its incidence with BBs, especially among patients initially taking drugs for diabetes.

### 4.1 A lower incidence of CDT with thiazides and ARBs than with BBs

We found that the incidence of CDT was lower with thiazides than with BBs in both the adherent patient population and the adherence-independent population. The results of this study support those of two systematic reviews conducted respectively by Fretheim et al. (2012) and Psaty et al. (2003). The findings of these two meta-analyses of randomized controlled trials (RCTs) indicated that diuretics were superior to BBs in preventing cardiovascular disease or cardiovascular events. Low-dose thiazides (generally daily doses of 12.5 mg–25 mg of chlorthalidone or hydrochlorothiazide) evidently reduce the risk of a CVD event (Psaty et al., 2003; Mishra, 2016; Wright et al., 2018). The incidence of CDT associated with ARBs was lower than that associated with BBs only in cohort 1. These results are similar to those of an RCT study (Dahlöf et al., 2002). Incidences of CDT associated with ACEIs and CCBs were similar than that of reference BBs. Fretheim et al. (2012) found that ACEIs were more effective than BBs in preventing myocardial infarction, but this meta-analysis of RCTs ranked the quality of their evidence as low. A striking finding from our previous investigation, which applied an ITT framework (Li et al., 2023), was that the incidence of CDT associated with CCBs was higher than that associated with BBs. This distinction may be attributed to PP analysis and consideration of time-varying confounding variables. Nevertheless, a common finding in both studies was that of a slightly lower survival rate of CCB users compared with those receiving BBs. Differing from our findings, those of the review by Ettehad et al. (2016) also showed that CCBs were superior to BBs in preventing major CVD events. However, only three trials were reviewed. Furthermore, CCBs and BBs were compared with placebos rather than with each other, there was considerable heterogeneity, and the evidence was deemed to be of low-to-moderate certainty (Wiysonge et al., 2017; Zhu et al., 2022). Consequently, their results may not have been reliable and could change in light of further trials. Overall, thiazides appear to be superior to BBs in their primary effect of preventing CVD events. This finding is consistent with those of earlier studies (Roush et al., 2014; Vögele et al., 2017; Li et al., 2023).

### 4.2 Subgroup and interaction effect

The incidence of CDT associated with thiazides and ARBs was lower than that associated with BBs in patients treated initially with diabetic drugs. After adjusting for adherence_180_, we still observed similar results. The correlation between diabetes and thiazides remains unclear (Verdecchia et al., 2005; Sica et al., 2011); diabetic patients in our study seemed to be more sensitive to thiazides. However, some bias may have arisen because of our classification of the diabetic subgroup according to the time-constant variable. The results for ARBs were consistent with the common guidelines, which can be verified from the baseline results.

These results showed that patients initially treated with drugs for diabetes accounted for the largest proportion of new ACEI and ARB users compared with users of the other three classes of monotherapies. All the results were aligned with global guidelines (Genootschap and en Innovatie, 2019; Visseren et al., 2021; WHO, 2021), which recommend ACEIs or ARBs as preferred monotherapies for diabetic patients. The reason could be that ARBs have therapeutic uses that extend well beyond antihypertensive effects since they may also improve insulin sensitivity (Izzo and Zion, 2011; Suksomboon et al., 2012; Abraham et al., 2015).

As there was no statistical difference in the interaction effects for these groups, we cannot conclude that diabetes drugs enhance the effect of thiazides relative to BBs. The incidence of CDT in male patients was lower with ARBs than with BBs, considering both the adherent population and all patients irrespective of adherence; the interaction effect also showed that male patients may have a positive effect to the comparison between ARBs and BBs compared with female patients. However, there is currently no relevant evidence to support this (Medina et al., 2020), so the choice of medication should be based on the practical condition of the patients. Considering the second-highest percentage of highly adherent ARB users, adherence did not seem to have any interaction effect on comparison between ARBs and BBs. Therefore, ARBs used as a monotherapy may improve adherence (Abraham et al., 2015).

### 4.3 Sensitivity analysis

In all four groups for which sensitivity analyses were performed, the results of the comparison between thiazides and BBs remained the same for both the 5-year and 10-year follow-up times. Therefore, it is likely that drug switches and add-ons did not significantly influence the results of the comparison of thiazides and BBs. However, we observed a higher incidence of CDT among adherent patients on CCBs who had switched drugs or had been prescribed a drug add-on compared with those on BBs. We think it could explain our conclusion in our previous paper (Li et al., 2023). Switching and adding on drugs may be confounding factors that influenced our results. The results of the sensitivity analyses may indicate that patients who switched or added other drugs had different hypertension risks or health conditions. It can be explained by the motivation of drug switching and drug add-on.

### 4.4 Strengths and limitations of the study

Our study had a number of strengths. First, it entailed a per-protocol analysis, which can reflect the effect of a real-world treatment strategy assigned throughout the follow-up period. Its findings complement those of a previous ITT study (Li et al., 2023) and strengthen the credibility of those results. Second, we used Cox regression with time-varying covariates, thus ensuring that the results were more consistent with a real-world situation. For example, some patients did not continually receive their prescriptions for comorbidities throughout the total follow-up time; rather, they received these prescriptions during different time periods. Third, we considered both time-constant and time-varying adherence, which is unique and can mitigate the bias between the compared adherence and outcomes. Fourth, we divided the population into two cohorts based on adherence_total_ and used the same analytical methods on both. Both approaches yielded similar results, indicating that our results are robust in relation to the chosen approach. Sensitivity analyses further demonstrated the robustness of our results. Finally, our results were representative and can provide a reference for the Netherlands, given that information on prescriptions was obtained from pharmacies located in different parts of this country.

Our study also had some limitations. First, we chose to follow up at 180-day intervals, using time-constant measures of drug adherence during this fixed-time interval, which biased the association between our CDT outcome and exposure (Bijlsma et al., 2016). For instance, patients who took their medicine irregularly may have had the same calculated adherence value as those who took it regularly. Second, we used time-varying variables for three drugs used to treat common comorbidities in the IPTW for adjustment, but we chose time-constant variables for the three initial drugs used for treating common comorbidities in the subgroup analysis, which may have generated some bias. Third, we did not consider the mortality induced by cardiovascular disease or other diseases because the IADB database only contains prescription information. Therefore, the sensitivity was reduced, and we missed some information. This random misclassification could have led us to underestimate the comparative effectiveness of the drugs. Finally, although we excluded patients with some other diseases who used the same types of antihypertensive monotherapy to prevent cardiovascular disease, potential unmeasured confounding factors may have played a role. For instance, CCBs are considered the first-line drug for treating Raynaud’s phenomenon (Rirash et al., 2017), and many CCBs are used as anti-angina and rate-lowering medications in atrial fibrillation, which may have led us to overestimate the relative effectiveness of CCBs compared with that of BBs.

## 5 Conclusion

Although indication bias cannot be ruled out completely, the findings of this real-world analysis suggest that long-term thiazide or ARB monotherapy appears to be associated with a lower incidence of CDT compared with BB monotherapy, notably among high-risk patients. Incidences of CDT associated with ACEIs and CCBs are comparably relative to those associated with BBs.

## Data Availability

The original contributions presented in the study are included in the article/Supplementary Material; further inquiries can be directed to the corresponding author.

## Abbreviations

ATC code, anatomical therapeutic chemical code; ACEIs, angiotensin-converting enzyme inhibitors; ARBs, angiotensin II receptor blockers; BBs, beta-blockers; CCBs, calcium channel blockers; CDT, cardiac drug therapy; CVD, cardiovascular diseases; COPD, chronic obstructive pulmonary disease; CI, confidence interval; HR, hazard ratio; IPTW, inverse probability treatment weighting; ITT, intention-to-treat; RA, rheumatoid arthritis; RCT, randomized controlled trial; sd, standard deviations.

## References

[B1] Abegaz T. M. Shehab A. Gebreyohannes E. A. Bhagavathula A. S. Elnour A. A. (2017). Nonadherence to antihypertensive drugs: a systematic review and meta-analysis. Med. Baltim. 96 (4), e5641. 10.1097/MD.0000000000005641 PMC5287944 28121920

[B2] Abraham H. M. White C. M. White W. B. (2015). The comparative efficacy and safety of the angiotensin receptor blockers in the management of hypertension and other cardiovascular diseases. Drug Saf. 38 (1), 33–54. 10.1007/s40264-014-0239-7 25416320 PMC4303500

[B3] Alfian S. D. Denig P. Coelho A. Hak E. (2019). Pharmacy-based predictors of non-adherence, non-persistence and reinitiation of antihypertensive drugs among patients on oral diabetes drugs in The Netherlands. PLoS One 14 (11), e0225390. 10.1371/journal.pone.0225390 31730627 PMC6857926

[B4] Bijlsma M. J. Janssen F. Hak E. (2016). Estimating time-varying drug adherence using electronic records: extending the proportion of days covered (PDC) method. Pharmacoepidemiol Drug Saf. 25 (3), 325–332. 10.1002/pds.3935 26687394

[B5] Black H. R. (1998). Antihypertensive therapy and cardiovascular disease. Impact of effective therapy on disease progression. Am. J. Hypertens. 11 (1 Pt 2), 3S–8s. 10.1016/s0895-7061(97)00422-6 9503100

[B6] Corrao G. Parodi A. Nicotra F. Zambon A. Merlino L. Cesana G. (2011). Better compliance to antihypertensive medications reduces cardiovascular risk. J. Hypertens. 29 (3), 610–618. 10.1097/HJH.0b013e328342ca97 21157368

[B7] Dahlöf B. Devereux R. B. Kjeldsen S. E. Julius S. Beevers G. de Faire U. (2002). Cardiovascular morbidity and mortality in the Losartan Intervention for Endpoint reduction in hypertension study (LIFE): a randomised trial against atenolol. Lancet 359 (9311), 995–1003. 10.1016/S0140-6736(02)08089-3 11937178

[B8] Ettehad D. Emdin C. A. Kiran A. Anderson S. G. Callender T. Emberson J. (2016). Blood pressure lowering for prevention of cardiovascular disease and death: a systematic review and meta-analysis. Lancet 387 (10022), 957–967. 10.1016/S0140-6736(15)01225-8 26724178

[B9] Fretheim A. Odgaard-Jensen J. Brørs O. Madsen S. Njølstad I. Norheim O. F. (2012). Comparative effectiveness of antihypertensive medication for primary prevention of cardiovascular disease: systematic review and multiple treatments meta-analysis. BMC Med. 10, 33. 10.1186/1741-7015-10-33 22480336 PMC3354999

[B10] Genootschap N. H. en Innovatie K. (2019) Praktische Handleiding bij de NHG-Standaard CVRM (2019). Nederlands: Huisartsen Genootschap.

[B11] Izzo J. L. Jr. Zion A. S. (2011). Value of Angiotensin receptor blocker therapy in diabetes. J. Clin. Hypertens. (Greenwich) 13 (4), 290–295. 10.1111/j.1751-7176.2011.00447.x 21466628 PMC8673251

[B12] Li J. Zhang Z. Si S. Wang B. Xue F. (2021). Antihypertensive medication adherence and cardiovascular disease risk: a longitudinal cohort study. Atherosclerosis 320, 24–30. 10.1016/j.atherosclerosis.2021.01.005 33516044

[B13] Li X. Bijlsma M. J. Bos J. H. J. Schuiling-Veninga C. C. M. Hak E. (2023). Long-term comparative effectiveness of antihypertensive monotherapies in primary prevention of cardiovascular events: a population-based retrospective inception cohort study in The Netherlands. BMJ Open 13 (8), e068721. 10.1136/bmjopen-2022-068721 PMC10414115 37558444

[B14] Medina D. Mehay D. Arnold A. C. (2020). Sex differences in cardiovascular actions of the renin-angiotensin system. Clin. Auton. Res. 30 (5), 393–408. 10.1007/s10286-020-00720-2 32860555 PMC7572792

[B15] Messerli F. H. Williams B. Ritz E. (2007). Essential hypertension. Lancet 370 (9587), 591–603. 10.1016/S0140-6736(07)61299-9 17707755

[B16] Mishra S. (2016). Diuretics in primary hypertension - reloaded. Indian Heart J. 68 (5), 720–723. 10.1016/j.ihj.2016.08.013 27773415 PMC5079206

[B17] Nishimura R. Kato H. Kisanuki K. Oh A. Hiroi S. Onishi Y. (2019). Treatment patterns, persistence and adherence rates in patients with type 2 diabetes mellitus in Japan: a claims-based cohort study. BMJ Open 9 (3), e025806. 10.1136/bmjopen-2018-025806 PMC6429930 30826768

[B18] Pouwels K. B. Voorham J. Hak E. Denig P. (2016). Identification of major cardiovascular events in patients with diabetes using primary care data. BMC Health Serv. Res. 16, 110. 10.1186/s12913-016-1361-2 27038959 PMC4818875

[B19] Psaty B. M. Lumley T. Furberg C. D. Schellenbaum G. Pahor M. Alderman M. H. (2003). Health outcomes associated with various antihypertensive therapies used as first-line agents: a network meta-analysis. Jama 289 (19), 2534–2544. 10.1001/jama.289.19.2534 12759325

[B20] Rirash F. Tingey P. C. Harding S. E. Maxwell L. J. Tanjong Ghogomu E. Wells G. A. (2017). Calcium channel blockers for primary and secondary Raynaud's phenomenon. Cochrane Database Syst. Rev. 12 (12), Cd000467. 10.1002/14651858.CD000467.pub2 29237099 PMC6486273

[B21] Roush G. C. Kaur R. Ernst M. E. (2014). Diuretics: a review and update. J. Cardiovasc Pharmacol. Ther. 19 (1), 5–13. 10.1177/1074248413497257 24243991

[B22] Salazar M. R. (2021). Early adherence to antihypertensive drugs and long-term cardiovascular mortality in the "real world. J. Clin. Hypertens. (Greenwich) 23 (9), 1703–1705. 10.1111/jch.14319 34254421 PMC8678652

[B23] Shau W. Y. Lai C. L. Huang S. T. Chen S. T. Li J. Z. Fung S. (2019). Statin adherence and persistence on secondary prevention of cardiovascular disease in Taiwan. Heart Asia 11 (2), e011176. 10.1136/heartasia-2018-011176 31565075 PMC6743447

[B24] Sica D. A. Carter B. Cushman W. Hamm L. (2011). Thiazide and loop diuretics. J. Clin. Hypertens. (Greenwich) 13 (9), 639–643. 10.1111/j.1751-7176.2011.00512.x 21896142 PMC8108854

[B25] Suchard M. A. Schuemie M. J. Krumholz H. M. You S. C. Chen R. Pratt N. (2019). Comprehensive comparative effectiveness and safety of first-line antihypertensive drug classes: a systematic, multinational, large-scale analysis. Lancet 394 (10211), 1816–1826. 10.1016/S0140-6736(19)32317-7 31668726 PMC6924620

[B26] Suksomboon N. Poolsup N. Prasit T. (2012). Systematic review of the effect of telmisartan on insulin sensitivity in hypertensive patients with insulin resistance or diabetes. J. Clin. Pharm. Ther. 37 (3), 319–327. 10.1111/j.1365-2710.2011.01295.x 21848583

[B27] Therneau T. Crowson C. Atkinson E. (2017). Using time dependent covariates and time dependent coefficients in the cox model. Surviv. Vignettes 2 (3), 1–25.

[B28] van der Wal W. M. Geskus R. B. (2011). Ipw: an R package for inverse probability weighting. J. Stat. Softw. 43 (13), 1–23. 10.18637/jss.v043.i13

[B29] Verdecchia P. Angeli F. Reboldi G. P. Gattobigio R. (2005). New-onset diabetes in treated hypertensive patients. Curr. Hypertens. Rep. 7 (3), 174–179. 10.1007/s11906-005-0006-3 15913490

[B30] Visser S. T. Schuiling-Veninga C. C. Bos J. H. de Jong-van den Berg L. T. Postma M. J. (2013). The population-based prescription database IADB.nl: its development, usefulness in outcomes research and challenges. Expert Rev. Pharmacoecon Outcomes Res. 13 (3), 285–292. 10.1586/erp.13.20 23763527

[B31] Visseren F. L. J. Mach F. Smulders Y. M. Carballo D. Koskinas K. C. Bäck M. (2021). 2021 ESC Guidelines on cardiovascular disease prevention in clinical practice. Eur. Heart J. 42 (34), 3227–3337. 10.1093/eurheartj/ehab484 34458905

[B32] Vögele A. Johansson T. Renom-Guiteras A. Reeves D. Rieckert A. Schlender L. (2017). Effectiveness and safety of beta blockers in the management of hypertension in older adults: a systematic review to help reduce inappropriate prescribing. BMC Geriatr. 17 (Suppl. 1), 224. 10.1186/s12877-017-0575-4 29047367 PMC5647554

[B33] WHO (2021) “Guideline for the pharmacological treatment of hypertension in adults,” in Translated from en by. Geneva: World Health Organization. 34495610

[B34] Whocc (2020). Methodology, W. C. C. f. D. S. high-ceiling diuretics. Available at: https://www.whocc.no/atc_ddd_index/?code=C03C (Accessed December 27, 2020).

[B35] Wiysonge C. S. Bradley H. A. Volmink J. Mayosi B. M. Opie L. H. (2017). Beta-blockers for hypertension. Cochrane Database Syst. Rev. 1 (1), Cd002003. 10.1002/14651858.CD002003.pub2 17253471

[B36] Wright J. M. Musini V. M. Gill R. (2018). First-line drugs for hypertension. Cochrane Database Syst. Rev. 4 (4), Cd001841. 10.1002/14651858.CD001841.pub3 29667175 PMC6513559

[B37] Zhu J. Chen N. Zhou M. Guo J. Zhu C. Zhou J. (2022). Calcium channel blockers versus other classes of drugs for hypertension. Cochrane Database Syst. Rev. 1 (1), Cd003654. 10.1002/14651858.cd003654.pub6 35000192 PMC8742884

