# Supplementary material for: Comparative effectiveness of antihypertensive monotherapies in primary prevention of cardiovascular events—a real-world longitudinal inception cohort study
Source: Front Pharmacol. 2024 Jun 5;15:1357567. doi: 10.3389/fphar.2024.1357567 (PMC11188318; doi:10.3389/fphar.2024.1357567)
Supplement: Supplementary file 1 [file DataSheet1.pdf]

Supplementary Material

Supplementary table 1 ATC codes used in our study

**All mentioned diseases and medications**	**ATC code**
**Anti-hypertensive drug monotherapies**	
ACEIs	C09A
ARBs	C09C
BBs	C07A
CCBs	C08C, C08D, C08E
Thiazides	C03AA
**Anti-hyperlipidemic drug monotherapies**	
HMG CoA reductase inhibitors	C10AA
Fibrates	C10AB
Bile acid sequestrants	C10AC
Nicotinic acid and derivatives	C10AD
Other lipid modifying agents	C10AX
**Antihypertensive drug fixed-dose combinations**	
Thiazides and potassium in combination	C03AB
Thiazides, combinations with psycholeptics and/or analgesics	C03AH
Thiazides, combinations with other drugs	C03AX
Calcium channel blockers and diuretics	C08G
Angiotensin converting enzyme inhibitors and combinations	C09B
Angiotensin II receptor blockers and combinations	C09D
Beta blocking agents and thiazides	C07B

Beta blocking agents and other diuretics	C07C
Beta blocking agents, thiazides and other diuretics	C07D
Beta blocking agents and vasodilators	C07E
Beta blocking agents, other combinations	C07F
**Anti-hyperlipidemic drug fixed-dose combinations**	
HMG CoA reductase inhibitors in combination with other lipid modifying agents	C10BA
HMG CoA reductase inhibitors, other combinations	C10BX
**Secondary prevention**	
Platelet aggregation inhibitor	B01AC
Vitamin K antagonist	B01AA
Organic nitrate	C01DA
Other vasodilators used in cardiac diseases	C01DX
**Chronic, stable heart failure**	
High-ceiling diuretics	C03C
**Migraine**	
Triptan	N02C
**Adrenal disease**	
Phentolamine	C04AB01
Tolazoline	C04AB02
Anticorticosteroids	H02CA
Mifeprostone	G03XB
Metyrapone	V04CD
**Hyperparathyroidism**	

Calcium, combinations with vitamin D and/or other drugs	A12AX
Vitamin D and analogues	A11CC
**Thyroid problems**	
Thyroid hormones	H03AA
**Diabetes**	
Blood glucose lowering drugs	A10
**Rheumatoid arthritis**	
Methotrexate	L04AX03
Sulfasalazine	A07EC01
Leflunomide	L04AA13
Etanercept	L04AB01
Infliximab	L04AB02
Adalimumab	L04AB04
Golimumab	L04AB06
Abatacept	L04AA24
Anakinra	L04AC03
Tocilizumab	L04AC07
**Asthma / COPD**	
Inhaled steroids	R03BA, R03AK, R03AL

Abbreviations: ACEIs, Angiotensin converting enzyme inhibitors; ARBs, Angiotensin II receptor blockers; BBs, Beta-blockers; CCBs, Calcium channel blockers; COPD, Chronic obstructive pulmonary disease.

Supplementary table 2 Baseline characteristics for cohort 2 population who used antihypertensive drugs monotherapy

**Demographics**	**Total**<br><br>**N=33,427**	**ACEIs**<br><br>**N=7,189 (21.5)** [a]	**ARBs**<br><br>**N=2,591 (7.8)** [a]	**BBs**<br><br>**N=13,712 (41.0)** [a]	**CCBs**<br><br>**N=3,167 (9.5)** [a]	**Thiazides**<br><br>**N=6,768 (20.2)** [a]	***P*** [b]
	**n (%)**	**n (%)**	**n (%)**	**n (%)**	**n (%)**	**n (%)**	
**Average follow-up years** [c]	3.2±2.9	3.6±2.8	3.8±2.9	2.9±3.0	2.7±2.4	3.3±2.8	/
**Initial comorbidities drug use**							
Diabetes drug: yes	1,676(5.0)	996 (13.9)	182 (7.0)	211 (1.5)	74 (2.3)	213 (3.1)	<0.001
RA drug: yes	280 (0.8)	72 (1.0)	29 (1.1)	71 (0.5)	56 (1.8)	52 (0.8)	<0.001
Asthma/COPD drug: yes	2,495(7.5)	594 (8.3)	240 (9.3)	743 (5.4)	302 (9.5)	616 (9.1)	<0.001
**Adherence$_{total}$**							<0.001
Low (<50%)	1,449(4.3)	46 (0.6)	21 (0.8)	1,176(8.6)	91 (2.9)	115 (1.7)	
Intermediate (50%~80%)	3,846 (11.5)	662 (9.2)	242 (9.3)	1,794(13.1)	398 (12.6)	750 (11.1)	
High (≥80%)	28,132 (84.2)	6,481 (90.2)	2,328 (89.8)	10,742 (78.3)	2,678 (84.6)	5,903 (87.2)	

Notes: [a]Row percentage, others are all column percentage.

[b]P value: significance value of the Chi-squared test or anova test, which showed the difference of distribution of patients who used five anti-hypertensive monotherapies at baseline in different subgroups of covariates.

[c]Use mean ± standard deviations to describe average follow-up years.

Abbreviations: ACEIs, Angiotensin converting enzyme inhibitors; ARBs, Angiotensin II receptor blockers; BBs, Beta-blockers; CCBs, Calcium channel blockers; RA, Rheumatoid arthritis; COPD, Chronic obstructive pulmonary disease.

Supplementary table 3 Cox regression analysis of acute CDT in different subgroups of cohort 1

**Subgroups**	**Crude HR [a] (95% CI)**								**IPTW adjusted HR [b] (95% CI)**							
	**ACEIs vs BBs**	P for interaction	**ARBs vs BBs**	P for interaction	**CCBs vs BBs**	P for interaction	**Thiazides vs BBs**	P for interaction	**ACEIs vs BBs**	P for interaction	**ARBs vs BBs**	P for interaction	**CCBs vs BBs**	P for interaction	**Thiazides vs BBs**	P for interaction
**Sex**																
Male	0.86 (0.71,1.03)	0.283	0.59 (0.43,0.80)	0.008	0.86 (0.64,1.17)	0.033	0.79 (0.63,1.00)	0.632	0.78 (0.63,0.97)	0.202	0.57 (0.42,0.78)	0.011	0.85 (0.62,1.15)	0.040	0.78 (0.62,0.99)	0.595
Female	1.00 (0.80,1.23)	ref	1.03 (0.77,1.37)	ref	1.32 (1.00,1.74)	ref	0.84 (0.68,1.04)	ref	0.95 (0.76,1.19)	ref	0.99 (0.74,1.33)	ref	1.28 (0.96,1.69)	ref	0.84 (0.68,1.03)	ref
**Age(years)**																
18-39	0.93 (0.46,1.90)	ref	NA†	ref	2.43 (0.93,6.38)	ref	0.54 (0.13,2.30)	ref	0.79 (0.38,1.67)	ref	NA†	ref	2.26 (0.84,6.05)	ref	0.49 (0.11,2.12)	ref
40-69	0.88 (0.74,1.05)	0.884	0.75 (0.58,0.97)	/	0.90 (0.68,1.19)	0.052	0.59 (0.48,0.74)	0.919	0.83 (0.68,1.00)	0.912	0.72 (0.55,0.94)	/	0.87 (0.66,1.16)	0.068	0.59 (0.48,0.73)	0.823
≥70	1.00 (0.79,1.27)	0.848	0.80 (0.56,1.14)	/	1.00 (0.72,1.38)	0.092	0.76 (0.60,0.97)	0.666	0.95 (0.73,1.22)	0.657	0.78 (0.54,1.11)	/	0.98 (0.71,1.37)	0.125	0.75 (0.59,0.95)	0.595
**Initial diabetes drug use**																
Yes	0.68 (0.40,1.16)	0.272	0.43 (0.19,0.97)	0.150	0.94 (0.34,2.57)	0.794	0.33 (0.13,0.85)	0.060	0.65 (0.35,1.22)	0.248	0.43 (0.19,0.98)	0.160	0.96 (0.35,2.69)	0.872	0.32 (0.13,0.82)	0.053
No	0.92 (0.79,1.07)	ref	0.81 (0.65,1.01)	ref	1.10 (0.89,1.36)	ref	0.83 (0.71,0.97)	ref	0.94 (0.81,1.10)	ref	0.80 (0.65,1.00)	ref	1.08 (0.87,1.33)	ref	0.82 (0.70,0.96)	ref
**Initial RA drug use**																
Yes	0.55 (0.12,2.44)	0.385	NA†	/	1.35 (0.38,4.82)	0.639	0.25 (0.03,2.24)	0.252	0.42 (0.09,2.01)	0.277	NA†	/	1.47 (0.44,4.97)	0.590	0.22 (0.03,1.80)	0.208
No	1.00 (0.87,1.14)	ref	0.82 (0.67,1.01)	ref	1.08 (0.87,1.33)	ref	0.82 (0.70,0.95)	ref	0.94 (0.81,1.09)	ref	0.80 (0.64,0.98)	ref	1.06 (0.86,1.31)	ref	0.81 (0.69,0.95)	ref

**Initial asthma/COPD drug use**																
Yes	1.23 (0.77,1.95)	0.321	1.37 (0.76,2.47)	0.058	1.01 (0.53,1.92)	0.847	0.93 (0.57,1.53)	0.539	0.85 (0.46,1.58)	0.759	1.26 (0.69,2.29)	0.102	0.99 (0.52,1.88)	0.857	0.89 (0.54,1.46)	0.681
No	0.96 (0.83,1.11)	ref	0.75 (0.60,0.94)	ref	1.11 (0.89,1.37)	ref	0.79 (0.67,0.93)	ref	0.94 (0.81,1.09)	ref	0.74 (0.59,0.93)	ref	1.08 (0.87,1.35)	ref	0.79 (0.67,0.93)	ref
**Calendar-year periods**																
1996-2000	1.54 (1.07,2.22)	ref	1.26 (0.63,2.53)	ref	2.22 (1.31,3.78)	ref	1.01 (0.63,1.61)	ref	1.48 (1.01,2.17)	ref	1.14 (0.55,2.34)	ref	2.27 (1.34,3.83)	ref	0.98 (0.61,1.58)	ref
2000-2010	1.04 (0.85,1.25)	0.056	0.79 (0.59,1.06)	0.239	1.56 (1.14,2.11)	0.282	0.83 (0.67,1.02)	0.473	0.95 (0.78,1.17)	0.046	0.78 (0.58,1.05)	0.356	1.51 (1.11,2.06)	0.213	0.82 (0.67,1.01)	0.512
2010-2020	0.94 (0.73,1.20)	0.024	0.88 (0.63,1.24)	0.384	0.89 (0.64,1.24)	0.004	0.81 (0.61,1.07)	0.433	0.91 (0.68,1.22)	0.047	0.86 (0.61,1.22)	0.517	0.87 (0.62,1.21)	0.002	0.80 (0.60,1.05)	0.451

Notes: P for interaction: Cox regression model contain treatment, confounding variables and their interaction term.

[a]Cox regression model only contain treatment.

[b]Cox regression model contain treatment and IPTW adjusted weights.

ref: reference group.

†: Number of events too small for effect size calculation.

/: If the HR of the reference group or control group was NA, then we did not consider the P for interaction of the control group.

Abbreviations: CDT, Cardiac drug therapy; HR, Hazard ratio; CI, Confidence interval; IPTW, Inverse Probability Treatment Weighting; ACEIs, Angiotensin converting enzyme inhibitors; ARBs, Angiotensin II receptor blockers; BBs, Beta-blockers; CCBs, Calcium channel blockers; RA, Rheumatoid arthritis; COPD, Chronic obstructive pulmonary disease.

Supplementary table 4 Cox regression analysis of acute CDT in different subgroups of cohort 2

**Subgroups**	**Crude HR [a] (95% CI)**								**IPTW adjusted HR [b] (95% CI)**							
	**ACEIs vs BBs**	P for interaction	**ARBs vs BBs**	P for interaction	**CCBs vs BBs**	P for interaction	**Thiazides vs BBs**	P for interaction	**ACEIs vs BBs**	P for interaction	**ARBs vs BBs**	P for interaction	**CCBs vs BBs**	P for interaction	**Thiazides vs BBs**	P for interaction
**Sex**																
Male	0.85 (0.71,1.01)	0.127	0.59 (0.45,0.79)	0.004	0.88 (0.66,1.17)	0.045	0.79 (0.64,0.99)	0.710	0.80 (0.65,0.99)	0.078	0.60 (0.44,0.80)	0.003	0.86 (0.65,1.14)	0.045	0.79 (0.64,0.99)	0.727
Female	1.04 (0.85,1.27)	ref	1.05 (0.80,1.38)	ref	1.29 (0.99,1.69)	ref	0.83 (0.68,1.02)	ref	1.05 (0.84,1.30)	ref	1.12 (0.83,1.51)	ref	1.27 (0.97,1.66)	ref	0.83 (0.68,1.01)	ref
**Age(years)**																
18-39	0.77 (0.39,1.52)	ref	0.25 (0.03,1.81)	ref	1.76 (0.68,4.54)	ref	0.64 (0.20,2.10)	ref	0.58 (0.28,1.21)	ref	0.31 (0.04,2.26)	ref	1.56 (0.59,4.13)	ref	0.63 (0.19,2.10)	ref
40-69	0.90 (0.76,1.06)	0.650	0.75 (0.59,0.97)	0.272	0.91 (0.70,1.18)	0.188	0.62 (0.51,0.76)	0.963	0.87 (0.72,1.05)	0.293	0.75 (0.57,0.98)	0.382	0.88 (0.68,1.15)	0.267	0.62 (0.50,0.76)	0.989
≥70	0.98 (0.79,1.23)	0.487	0.80 (0.58,1.12)	0.248	0.98 (0.72,1.34)	0.255	0.70 (0.55,0.88)	0.901	0.95 (0.75,1.21)	0.208	0.86 (0.60,1.21)	0.318	0.98 (0.72,1.33)	0.374	0.69 (0.55,0.87)	0.876
**Initial diabetes drug use**																
Yes	0.69 (0.42,1.15)	0.299	0.38 (0.17,0.84)	0.063	1.03 (0.41,2.60)	0.951	0.34 (0.14,0.82)	0.052	0.68 (0.36,1.29)	0.278	0.36 (0.16,0.82)	0.044	1.02 (0.39,2.64)	0.958	0.35 (0.15,0.84)	0.064
No	0.91 (0.79,1.05)	ref	0.83 (0.68,1.02)	ref	1.09 (0.90,1.34)	ref	0.82 (0.71,0.95)	ref	0.98 (0.85,1.14)	ref	0.88 (0.71,1.09)	ref	1.08 (0.88,1.32)	ref	0.82 (0.71,0.96)	ref
**Initial RA drug use**																
Yes	0.56 (0.13,2.52)	0.403	NA†	/	1.61 (0.47,5.52)	0.454	0.25 (0.03,2.25)	0.254	0.41 (0.09,1.99)	0.248	NA†	/	1.70 (0.52,5.53)	0.450	0.21 (0.03,1.70)	0.199
No	1.00 (0.88,1.15)	ref	0.83 (0.68,1.02)	ref	1.07 (0.87,1.30)	ref	0.81 (0.70,0.94)	ref	0.99 (0.85,1.14)	ref	0.86 (0.70,1.06)	ref	1.07 (0.87,1.30)	ref	0.81 (0.70,0.94)	ref

**Initial asthma/COPD drug use**																
Yes	1.33 (0.85,2.07)	0.179	1.25 (0.70,2.25)	0.124	1.05 (0.57,1.93)	0.955	0.98 (0.61,1.57)	0.386	0.96 (0.51,1.81)	0.945	1.07 (0.59,1.95)	0.434	1.01 (0.55,1.86)	0.909	0.93 (0.58,1.50)	0.526
No	0.96 (0.84,1.11)	ref	0.77 (0.63,0.96)	ref	1.10 (0.89,1.35)	ref	0.78 (0.66,0.91)	ref	0.98 (0.85,1.13)	ref	0.83 (0.66,1.04)	ref	1.08 (0.88,1.33)	ref	0.79 (0.67,0.92)	ref
**Calendar-year periods**																
1996-2000	1.70 (1.20,2.41)	ref	1.29 (0.65,2.58)	ref	2.37 (1.44,3.89)	ref	0.98 (0.62,1.56)	ref	1.72 (1.19,2.47)	ref	1.19 (0.58,2.45)	ref	2.44 (1.49,3.99)	ref	0.96 (0.61,1.52)	ref
2000-2010	1.05 (0.87,1.25)	0.015	0.79 (0.60,1.04)	0.206	1.53 (1.14,2.04)	0.146	0.83 (0.68,1.01)	0.508	1.02 (0.84,1.24)	0.015	0.84 (0.62,1.13)	0.401	1.47 (1.09,1.97)	0.089	0.83 (0.68,1.00)	0.568
2010-2020	0.91 (0.72,1.15)	0.003	0.91 (0.66,1.26)	0.392	0.86 (0.63,1.19)	0.001	0.79 (0.61,1.03)	0.424	0.90 (0.68,1.20)	0.007	0.93 (0.67,1.30)	0.573	0.86 (0.62,1.17)	<0.001	0.79 (0.61,1.03)	0.482
**Adherence$_{total}$**																
Low (<50%)	4.77 (1.19,19.11)	ref	2.08 (0.25,17.34)	ref	7.89 (1.85,33.75)	ref	NA†	ref	5.82 (1.32,25.70)	ref	3.29 (0.36,29.97)	ref	8.10 (2.53,25.95)	ref	NA†	ref
Intermediate (50%~80%)	1.06 (0.68,1.64)	0.024	0.94 (0.50,1.80)	0.408	0.83 (0.43,1.63)	0.007	0.80 (0.49,1.32)	/	1.15 (0.71,1.86)	0.030	1.13 (0.56,2.26)	0.301	0.82 (0.41,1.63)	0.002	0.80 (0.49,1.32)	/
High (≥80%)	0.99 (0.86,1.14)	0.014	0.81 (0.66,1.00)	0.319	1.10 (0.90,1.36)	0.009	0.81 (0.69,0.95)	/	0.95 (0.81,1.10)	0.012	0.80 (0.65,1.00)	0.170	1.08 (0.88,1.33)	0.001	0.81 (0.69,0.94)	/

Notes: P for interaction: Cox regression model contain treatment, confounding variables and their interaction term.

[a]Cox regression model only contain treatment.

[b]Cox regression model contain treatment and IPTW adjusted weights.

ref: reference group.

†: Number of events too small for effect size calculation.

/: If the HR of the reference group or control group was NA, then we did not consider the P for interaction of the control group.

Abbreviations: CDT, Cardiac drug therapy; HR, Hazard ratio; CI, Confidence interval; IPTW, Inverse Probability Treatment Weighting; ACEIs, Angiotensin converting enzyme inhibitors; ARBs, Angiotensin II receptor blockers; BBs, Beta-blockers; CCBs, Calcium channel blockers; RA, Rheumatoid arthritis; COPD, Chronic obstructive pulmonary disease.

Supplementary table 5 Cox regression analysis of acute CDT in cohort 3 and cohort 4

Acute CDT																
	**Cohort 3 (10-year)**				**Cohort 3 (5-year)**				**Cohort 4 (10-year)**				**Cohort 4 (5-year)**			
**Anti-hypertensive monotherapies**	**Crude HR[a] (95%CI)**	***P***	**IPTW adjusted[b] HR (95%CI)**	***P***	**Crude HR[a] (95%CI)**	***P***	**IPTW adjusted[b] HR (95%CI)**	***P***	**Crude HR[a] (95%CI)**	***P***	**IPTW adjusted[b] HR (95%CI)**	***P***	**Crude HR[a] (95%CI)**	***P***	**IPTW adjusted[b] HR (95%CI)**	***P***
**Reference: BBs**																
**Exposure**																
ACEIs	0.91 (0.74,1.11)	0.345	0.83 (0.66,1.03)	0.086	0.90 (0.72,1.12)	0.354	0.83 (0.65,1.06)	0.130	0.88 (0.72,1.07)	0.191	0.82 (0.66,1.02)	0.073	0.90 (0.73,1.11)	0.324	0.83 (0.66,1.05)	0.123
ARBs	0.79 (0.58,1.08)	0.145	0.76 (0.56,1.04)	0.091	0.74 (0.53,1.04)	0.082	0.72 (0.51,1.01)	0.060	0.82 (0.61,1.09)	0.171	0.85 (0.61,1.17)	0.319	0.80 (0.58,1.09)	0.156	0.85 (0.60,1.20)	0.346
CCBs	1.00 (0.74,1.35)	0.985	0.98 (0.72,1.33)	0.879	1.18 (0.88,1.58)	0.282	1.16 (0.86,1.56)	0.331	0.97 (0.73,1.29)	0.841	0.96 (0.72,1.28)	0.775	1.17 (0.89,1.55)	0.269	1.16 (0.88,1.54)	0.297
Thiazides	0.70 (0.54,0.89)	0.004	0.70 (0.54,0.90)	0.005	0.60 (0.45,0.78)	<0.001	0.59 (0.45,0.78)	<0.001	0.71 (0.56,0.89)	0.004	0.72 (0.57,0.91)	0.005	0.61 (0.48,0.79)	<0.001	0.62 (0.48,0.80)	<0.001

Notes: [a]Cox regression model only contain treatment.

[b]Cox regression model contain treatment and IPTW adjusted weights.

Abbreviations: CDT, Cardiac drug therapy; HR, Hazard ratio; CI, Confidence interval; IPTW, Inverse Probability Treatment Weighting; ACEIs, Angiotensin converting enzyme inhibitors; ARBs, Angiotensin II receptor blockers; BBs, Beta-blockers; CCBs, Calcium channel blockers.

Supplementary table 6 Cox regression analysis of acute CDT in cohort 5 and cohort 6

Acute CDT																
	Cohort 5 (10-year)				Cohort 5 (5-year)				Cohort 6 (10-year)				Cohort 6 (5-year)			
**Anti-hypertensive monotherapies**	**Crude HR[a] (95%CI)**	***P***	**IPTW adjusted[b] HR (95%CI)**	***P***	**Crude HR[a] (95%CI)**	***P***	**IPTW adjusted[b] HR (95%CI)**	***P***	**Crude HR[a] (95%CI)**	***P***	**IPTW adjusted[b] HR (95%CI)**	***P***	**Crude HR[a] (95%CI)**	***P***	**IPTW adjusted[b] HR (95%CI)**	***P***
Reference:BBs																
Exposure																
ACEIs	1.07 (0.89,1.29)	0.462	1.07 (0.88,1.29)	0.511	0.89 (0.70,1.13)	0.340	0.88 (0.68,1.13)	0.313	1.10 (0.92,1.31)	0.296	1.10 (0.91,1.32)	0.324	0.93 (0.74,1.16)	0.508	0.92 (0.73,1.17)	0.503
ARBs	0.79 (0.59,1.05)	0.099	0.78 (0.59,1.04)	0.090	0.80 (0.56,1.15)	0.235	0.79 (0.55,1.13)	0.198	0.78 (0.59,1.02)	0.069	0.78 (0.59,1.03)	0.079	0.80 (0.57,1.12)	0.202	0.80 (0.57,1.13)	0.201
CCBs	1.39 (1.05,1.84)	0.020	1.35 (1.02,1.79)	0.036	1.01 (0.68,1.48)	0.974	0.96 (0.65,1.41)	0.822	1.44 (1.11,1.88)	0.007	1.40 (1.07,1.83)	0.014	1.11 (0.78,1.58)	0.559	1.05 (0.74,1.50)	0.780
Thiazides	0.78 (0.64,0.95)	0.015	0.77 (0.63,0.94)	0.010	0.73 (0.56,0.94)	0.016	0.72 (0.55,0.93)	0.012	0.75 (0.62,0.92)	0.004	0.74 (0.61,0.90)	0.003	0.68 (0.53,0.87)	0.002	0.67 (0.52,0.86)	0.002

Notes: [a]Cox regression model only contain treatment.

[b]Cox regression model contain treatment and IPTW adjusted weights.

Abbreviations: CDT, Cardiac drug therapy; HR, Hazard ratio; CI, Confidence interval; IPTW, Inverse Probability Treatment Weighting; ACEIs, Angiotensin converting enzyme inhibitors; ARBs, Angiotensin II receptor blockers; BBs, Beta-blockers; CCBs, Calcium channel blockers.

Supplementary Figure 1 Survival curves for acute CDT in cohort 3/4 patients treated with 4 types of anti-hypertensive monotherapies compared with BBs in 10-year and 5-year of time before and after IPW. (A1) ACEIs vs BBs (cohort3, 10-year follow up), (A2) ACEIs vs BBs (cohort3, 5-year follow up), (B1) ARBs vs BBs (cohort3, 10-year follow up), (B2) ARBs vs BBs (cohort3, 5-year follow up), (C1) CCBs vs BBs (cohort3, 10-year follow up), (C2) CCBs vs BBs (cohort3, 5-year follow up), (D1) Thiazides vs BBs (cohort3, 10-year follow up), (D2) Thiazides vs BBs (cohort3, 5-year follow up), (E1) ACEIs vs BBs (cohort4, 10-year follow up), (E2) ACEIs vs BBs (cohort4, 5-year follow up), (F1) ARBs vs BBs (cohort4, 10-year follow up), (F2) ARBs vs BBs (cohort4, 5-year follow up), (G1) CCBs vs BBs (cohort4, 10-year follow up), (G2) CCBs vs BBs (cohort4, 5-year follow up), (H1)Thiazides vs BBs (cohort4, 10-year follow up), (H2) Thiazides vs BBs (cohort4, 5-year follow up).

Notes: Before: time varying Cox regression before IPTW adjustment; After: time varying Cox regression after IPTW adjustment.

Abbreviations: CDT, Cardiac drug therapy; ACEIs, Angiotensin converting enzyme inhibitors; ARBs, Angiotensin II receptor blockers; BBs, Beta-blockers; CCBs, Calcium channel blockers; IPTW, Inverse Probability Treatment Weighting.

Supplementary Figure 2 Survival curves for acute CDT in cohort 5/6 patients treated with 4 types of anti-hypertensive monotherapies compared with BBs in 10-year and 5-year of time before and after IPW. (A1) ACEIs vs BBs (cohort5, 10-year follow up), (A2) ACEIs vs BBs (cohort5, 5-year follow up), (B1) ARBs vs BBs (cohort5, 10-year follow up), (B2) ARBs vs BBs (cohort5, 5-year follow up), (C1) CCBs vs BBs (cohort5, 10-year follow up), (C2) CCBs vs BBs (cohort5, 5-year follow up), (D1) Thiazides vs BBs (cohort5, 10-year follow up), (D2) Thiazides vs BBs (cohort5, 5-year follow up), (E1) ACEIs vs BBs (cohort6, 10-year follow up), (E2) ACEIs vs BBs (cohort6, 5-year follow up), (F1) ARBs vs BBs (cohort6, 10-year follow up), (F2) ARBs vs BBs (cohort6, 5-year follow up), (G1) CCBs vs BBs (cohort6, 10-year follow up), (G2) CCBs vs BBs (cohort6, 5-year follow up), (H1)Thiazides vs BBs (cohort6, 10-year follow up), (H2) Thiazides vs BBs (cohort6, 5-year follow up).

Notes: Before: time varying Cox regression before IPTW adjustment; After: time varying Cox regression after IPTW adjustment.

Abbreviations: CDT, Cardiac drug therapy; ACEIs, Angiotensin converting enzyme inhibitors; ARBs, Angiotensin II receptor blockers; BBs, Beta-blockers; CCBs, Calcium channel blockers; IPTW, Inverse Probability Treatment Weighting.

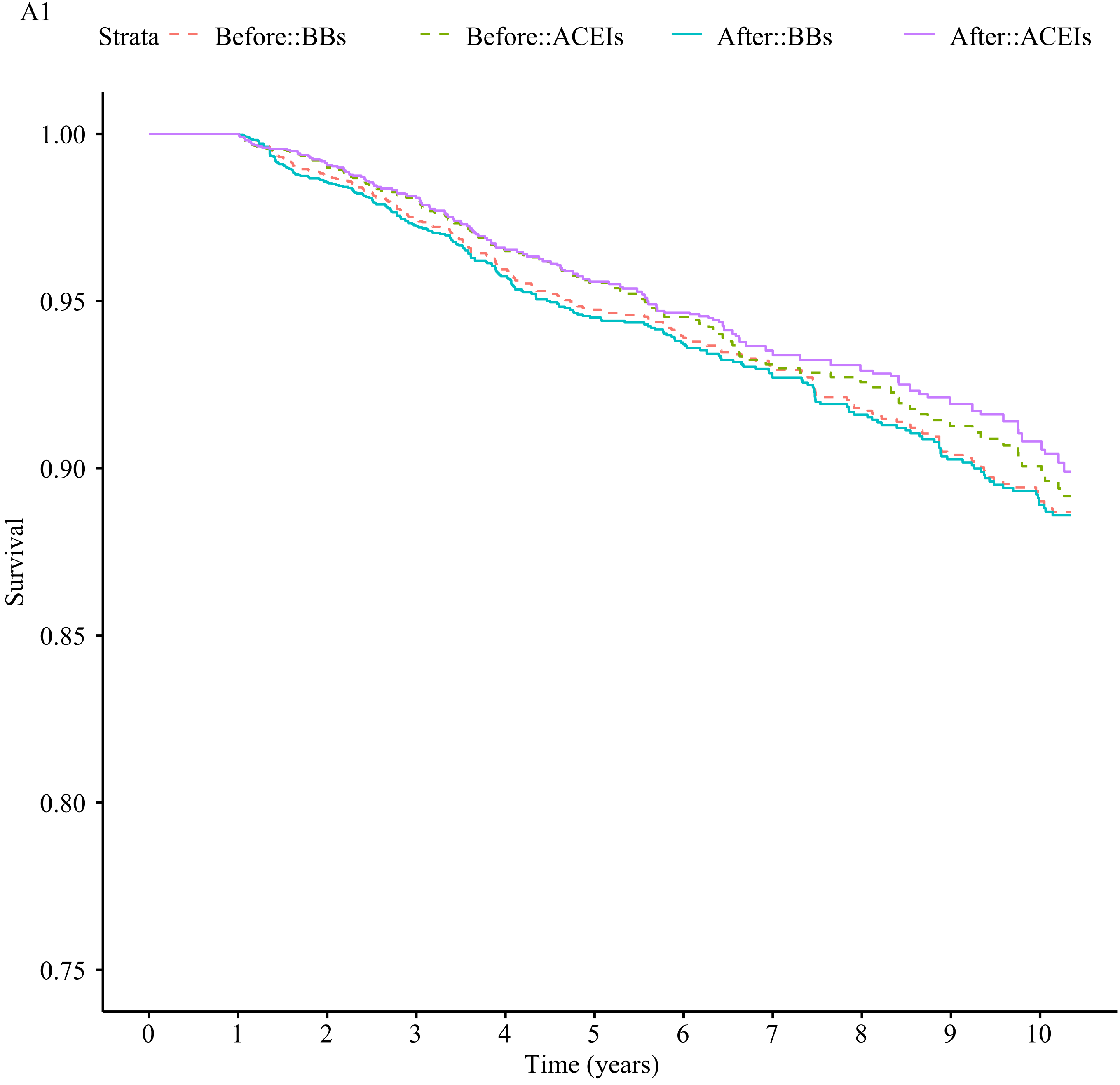
A1
Strata
Before::BBs
Before::ACEIs
After::BBs
After::ACEIs
Survival
1.00
0.95
0.90
0.85
0.80
0.75
0
1
2
3
4
5
6
7
8
9
10
Time (years)

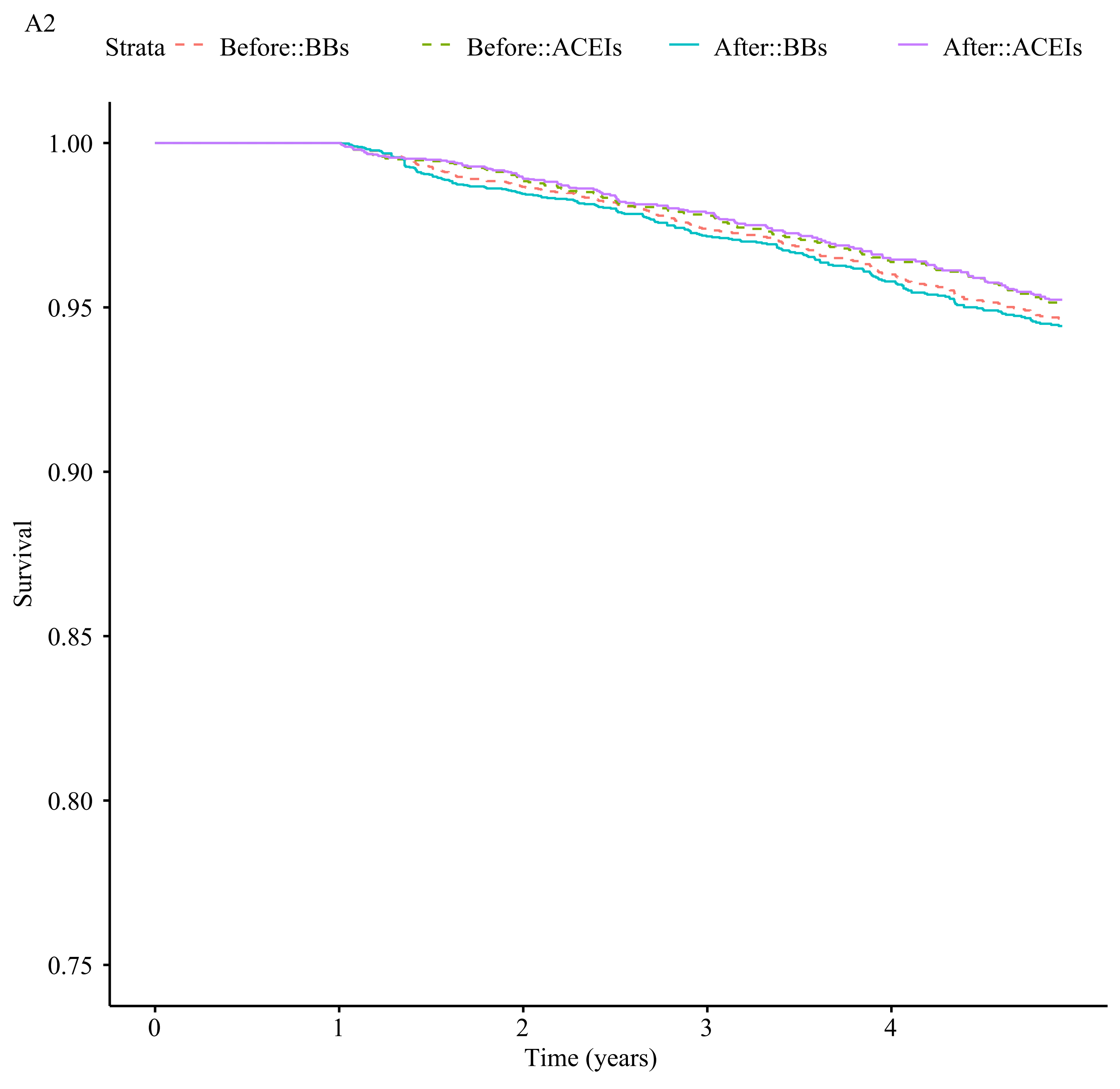
A2
Strata
Before::BBs
Before::ACEIs
After::BBs
After::ACEIs
1.00
0.95
0.90
0.85
0.80
0.75
Survival
0
1
2
3
4
Time (years)

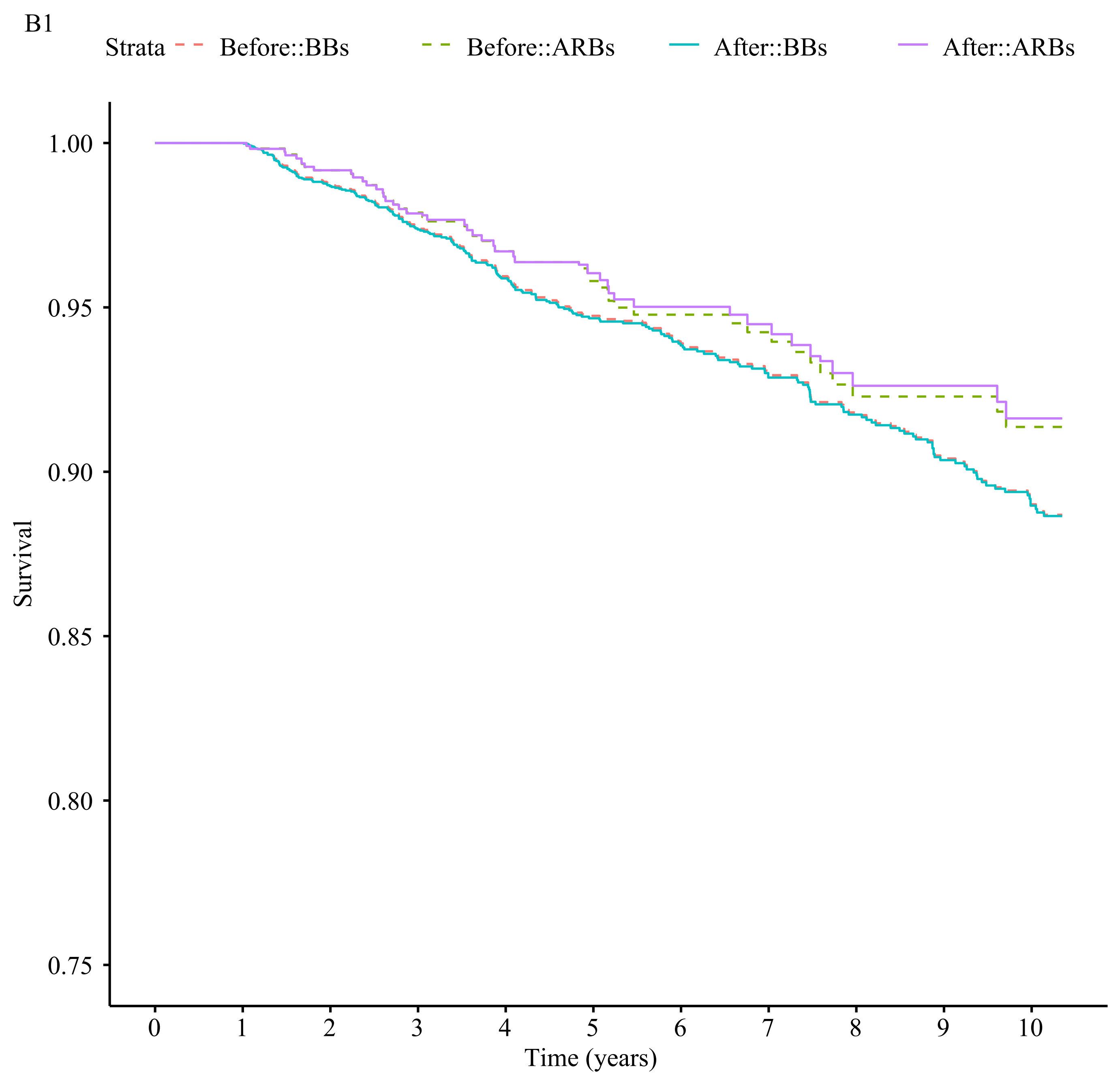
B1
Strata
Before::BBs
Before::ARBs
After::BBs
After::ARBs
Survival
1.00
0.95
0.90
0.85
0.80
0.75
0
1
2
3
4
5
6
7
8
9
10
Time (years)

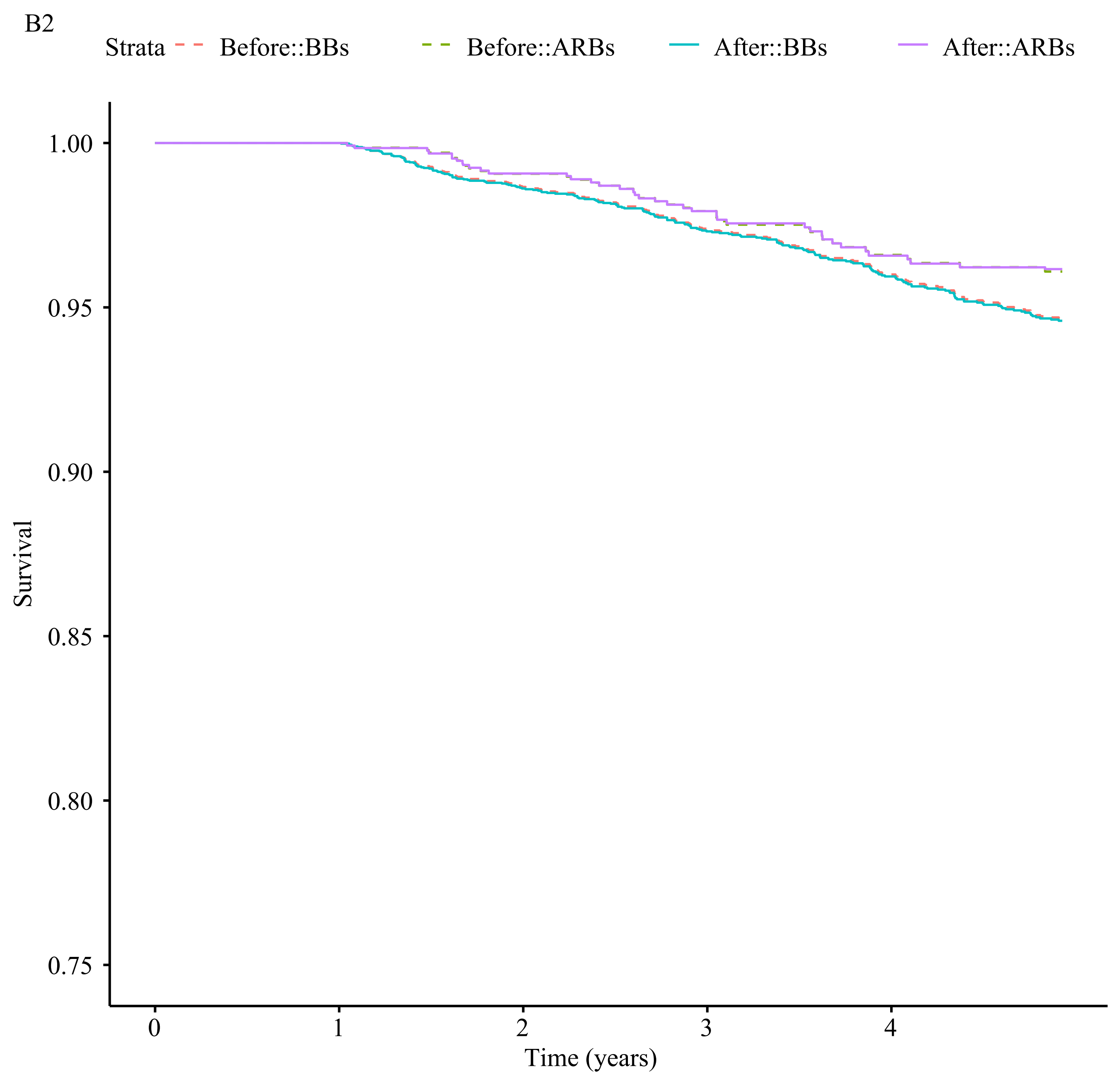

B2
Strata
Before::BBs
Before::ARBs
After::BBs
After::ARBs
1.00
0.95
0.90
0.85
0.80
0.75
Survival
0
1
2
3
4
Time (years)

C1

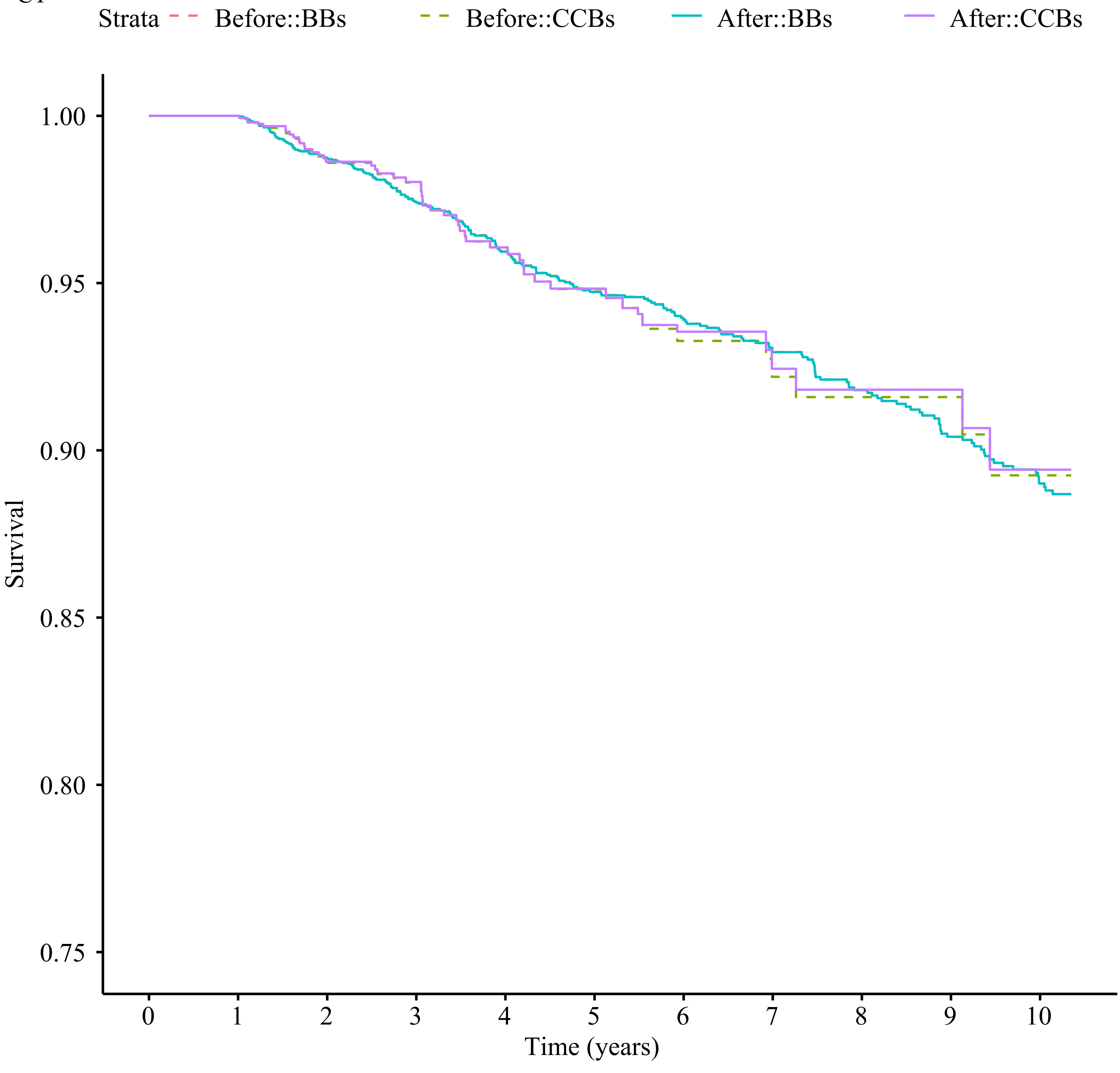

Strata
Before::BBs
Before::CCBs
After::BBs
After::CCBs
Survival
1.00
0.95
0.90
0.85
0.80
0.75
0
1
2
3
4
5
6
7
8
9
10
Time (years)

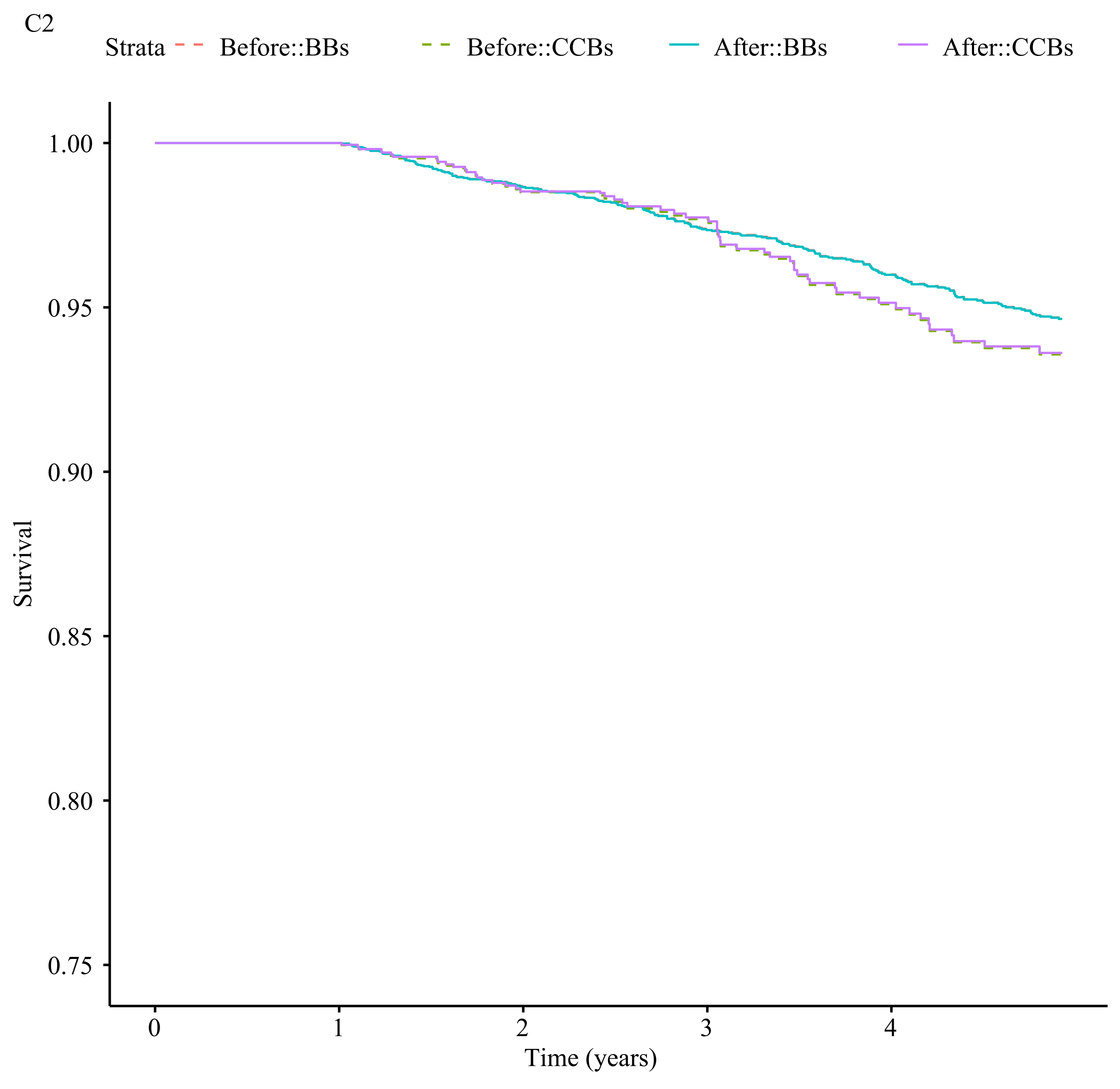

C2
Strata
Before::BBs
Before::CCBs
After::BBs
After::CCBs
Survival
1.00
0.95
0.90
0.85
0.80
0.75
0
1
2
3
4
Time (years)

D1

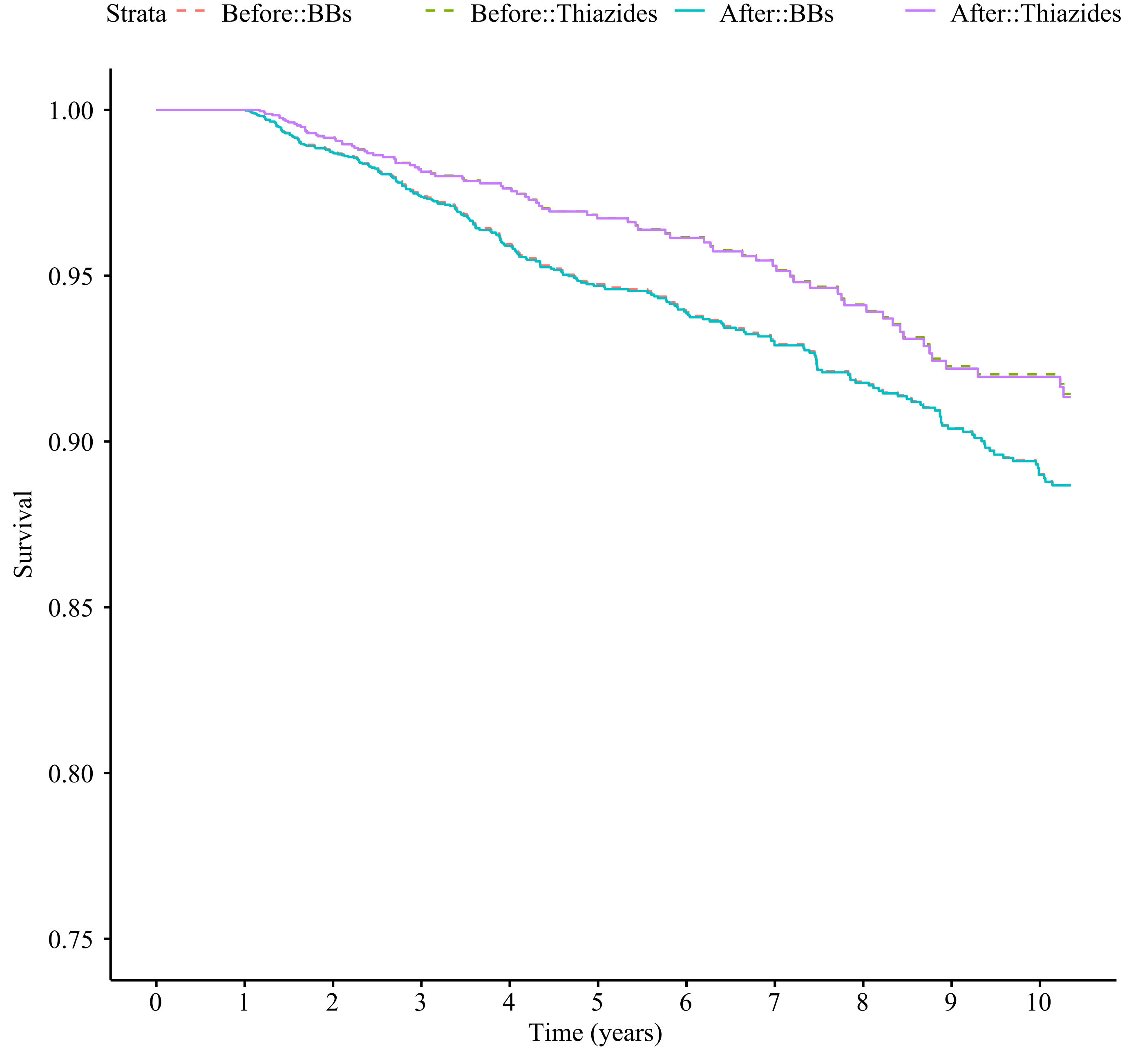

Strata
Before::BBs
Before::Thiazides
After::BBs
After::Thiazides
1.00
0.95
0.90
0.85
0.80
0.75
Survival
0
1
2
3
4
5
6
7
8
9
10
Time (years)

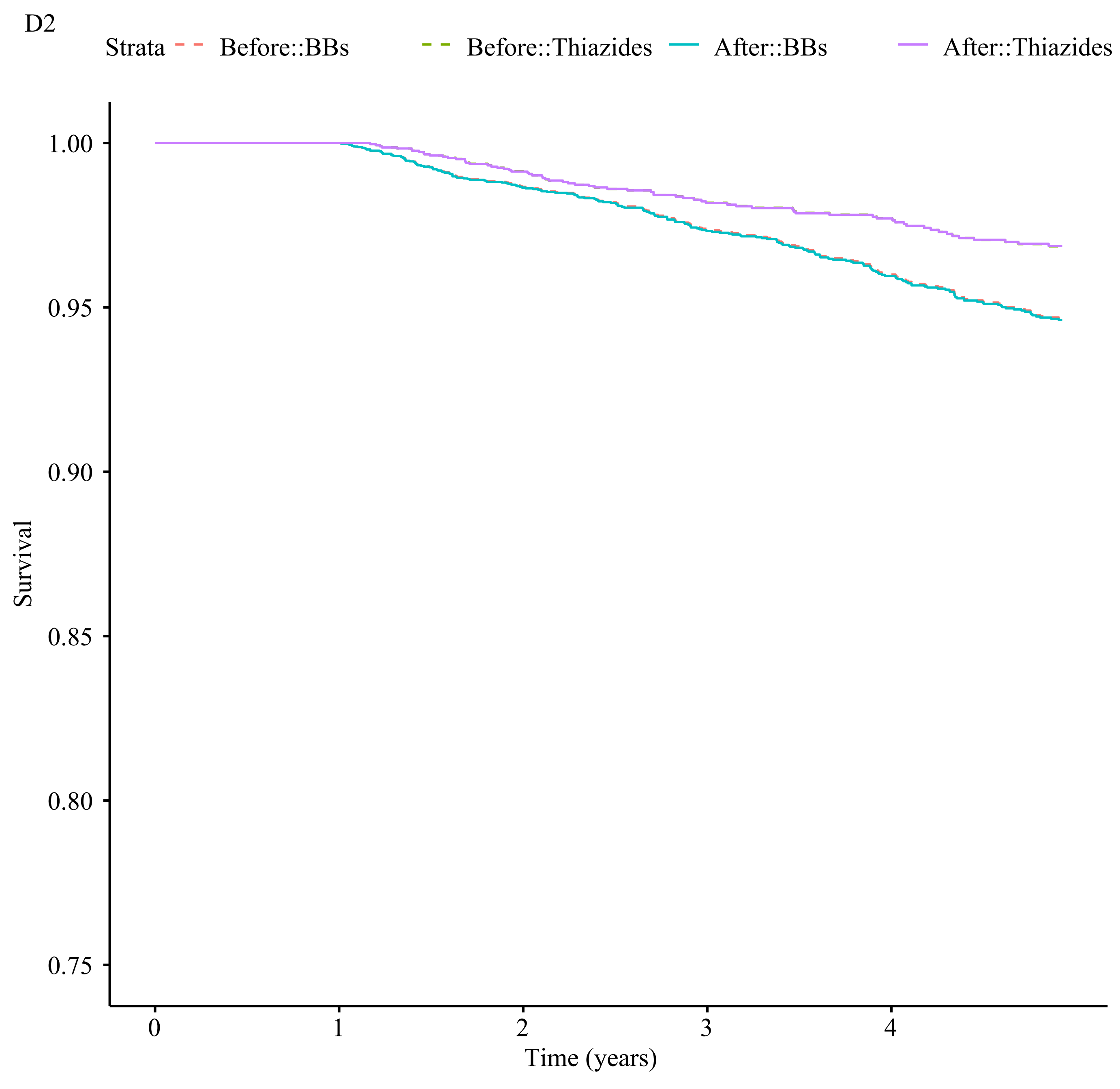
D2
Strata
Before::BBs
Before::Thiazides
After::BBs
After::Thiazides
Survival
1.00
0.95
0.90
0.85
0.80
0.75
0
1
2
3
4
Time (years)

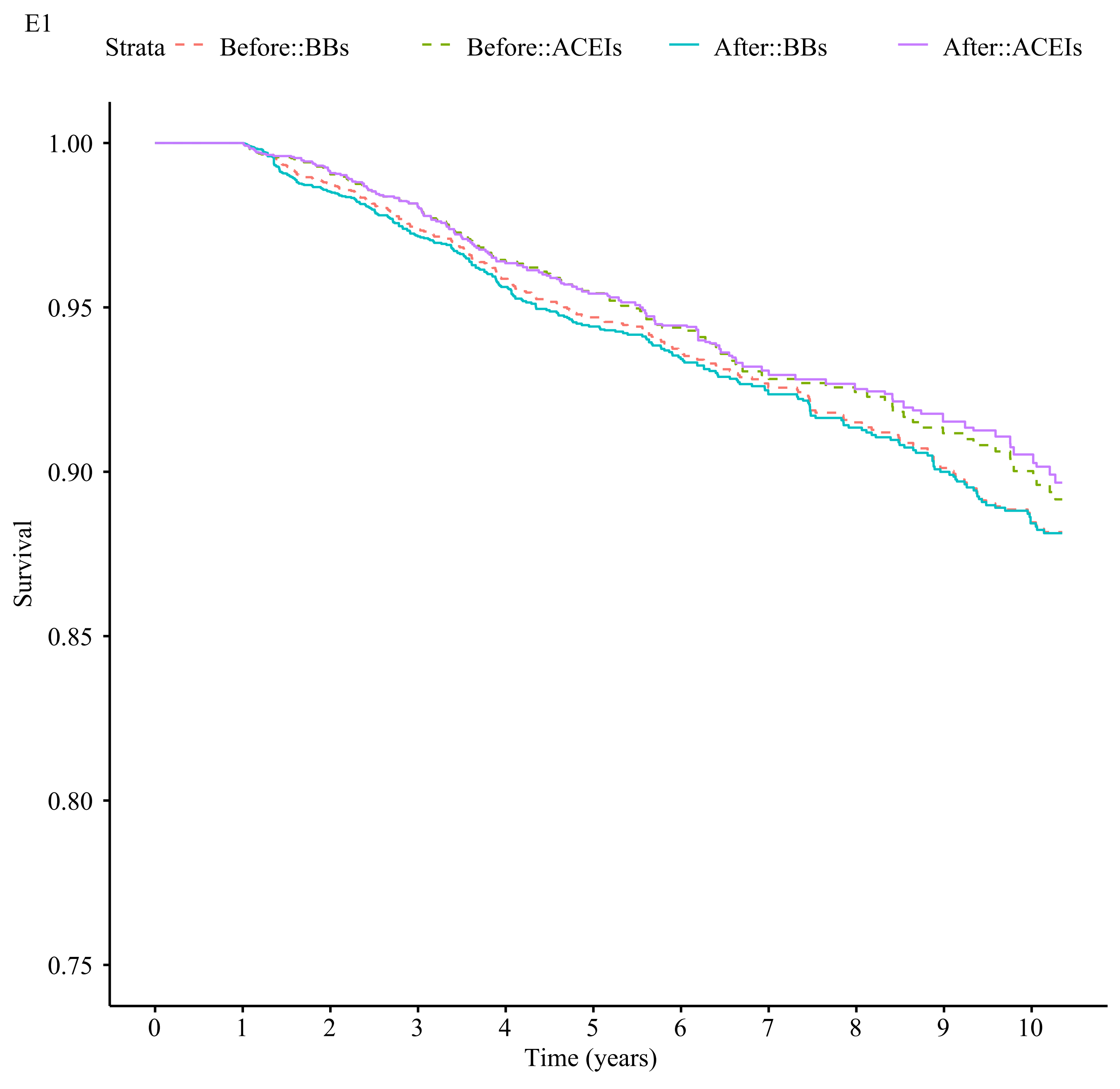
E1
Strata
Before::BBs
Before::ACEIs
After::BBs
After::ACEIs
Survival
1.00
0.95
0.90
0.85
0.80
0.75
0
1
2
3
4
5
6
7
8
9
10
Time (years)

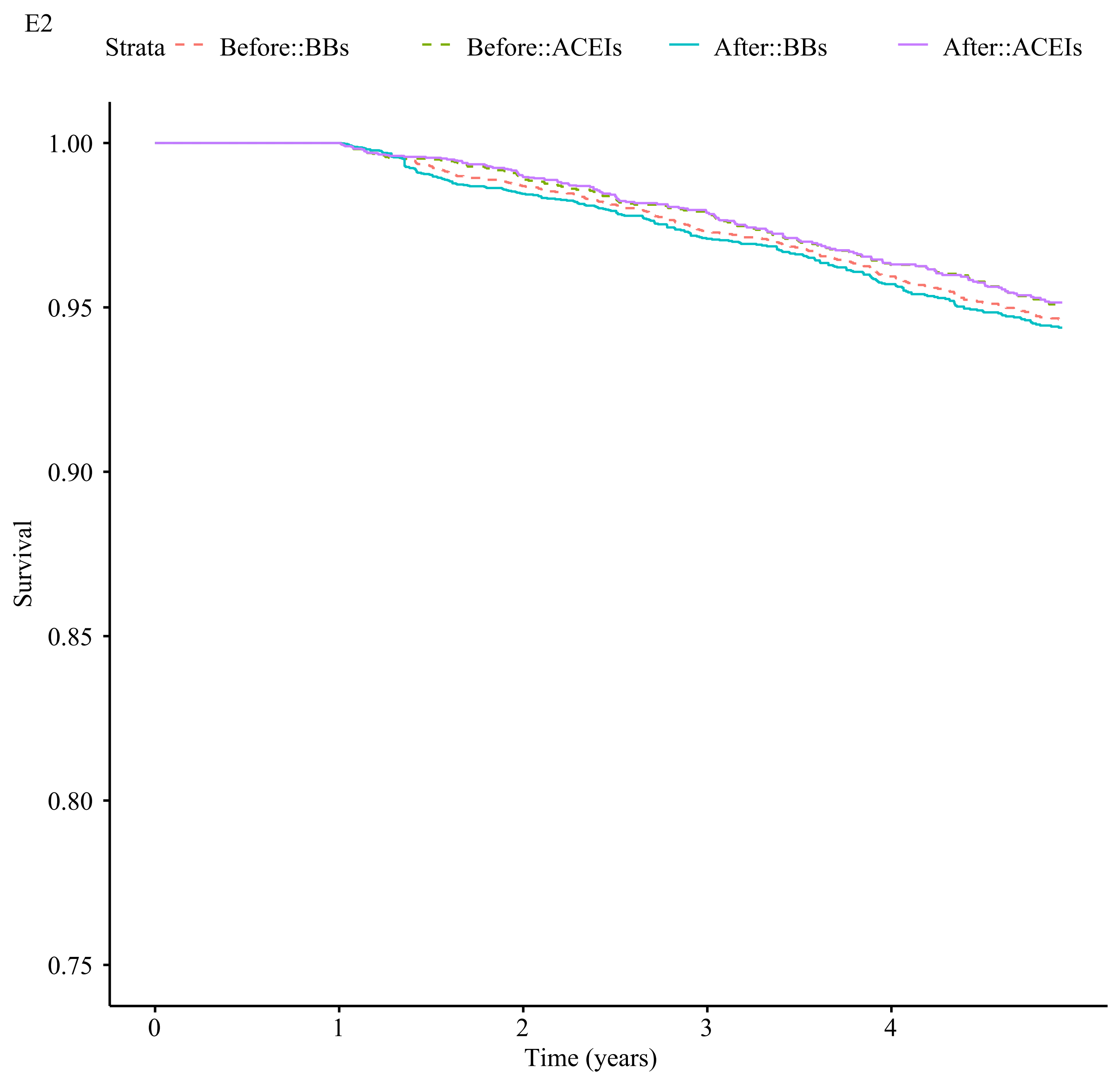
E2
Strata
Before::BBs
Before::ACEIs
After::BBs
After::ACEIs
1.00
0.95
0.90
0.85
0.80
0.75
Survival
0
1
2
3
4
Time (years)

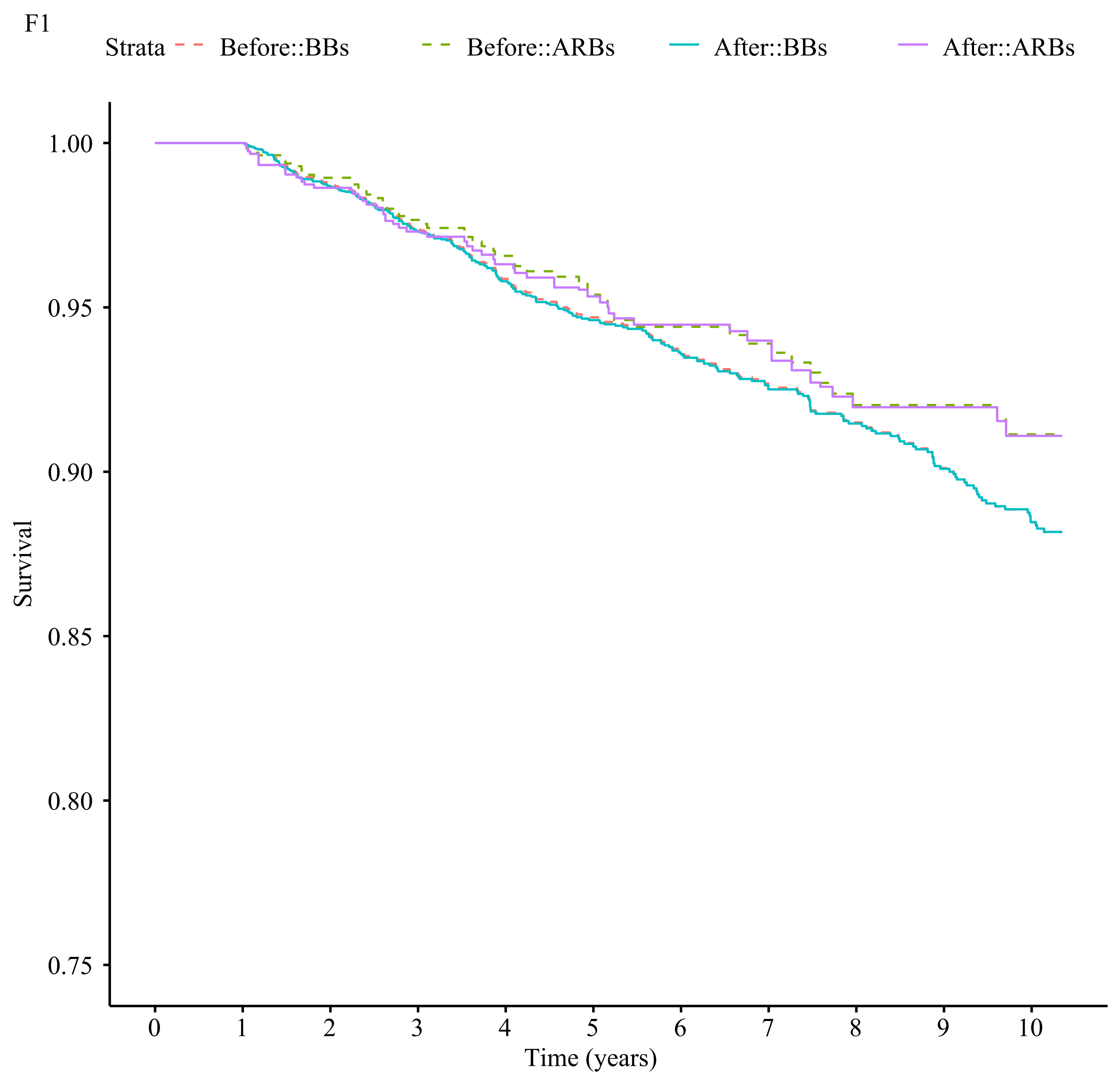
F1
Strata
Before::BBs
Before::ARBs
After::BBs
After::ARBs
Survival
1.00
0.95
0.90
0.85
0.80
0.75
0
1
2
3
4
5
6
7
8
9
10
Time (years)

F2

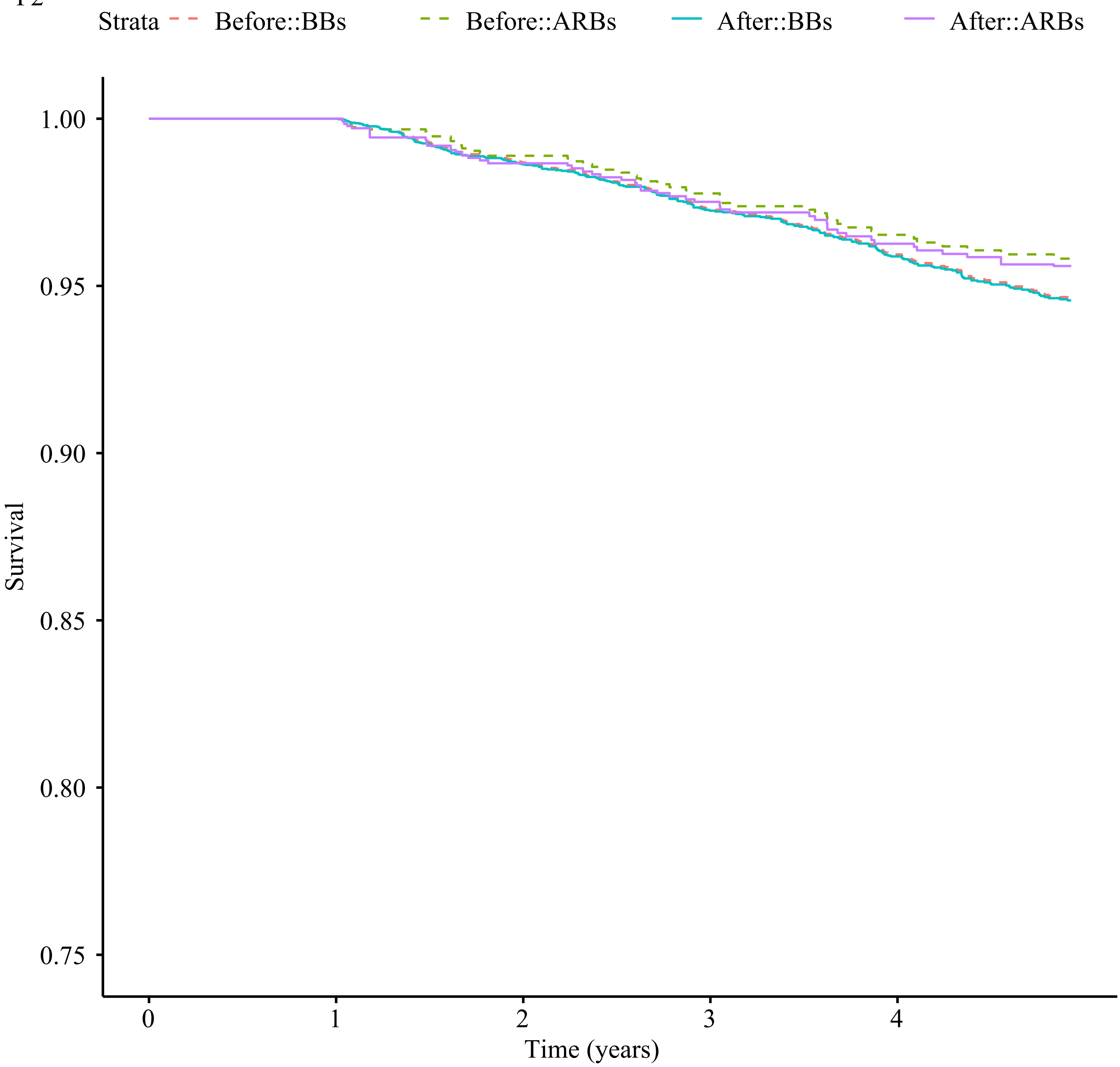
Strata
Before::BBs
Before::ARBs
After::BBs
After::ARBs
1.00
0.95
0.90
0.85
0.80
0.75
Survival
0
1
2
3
4
Time (years)

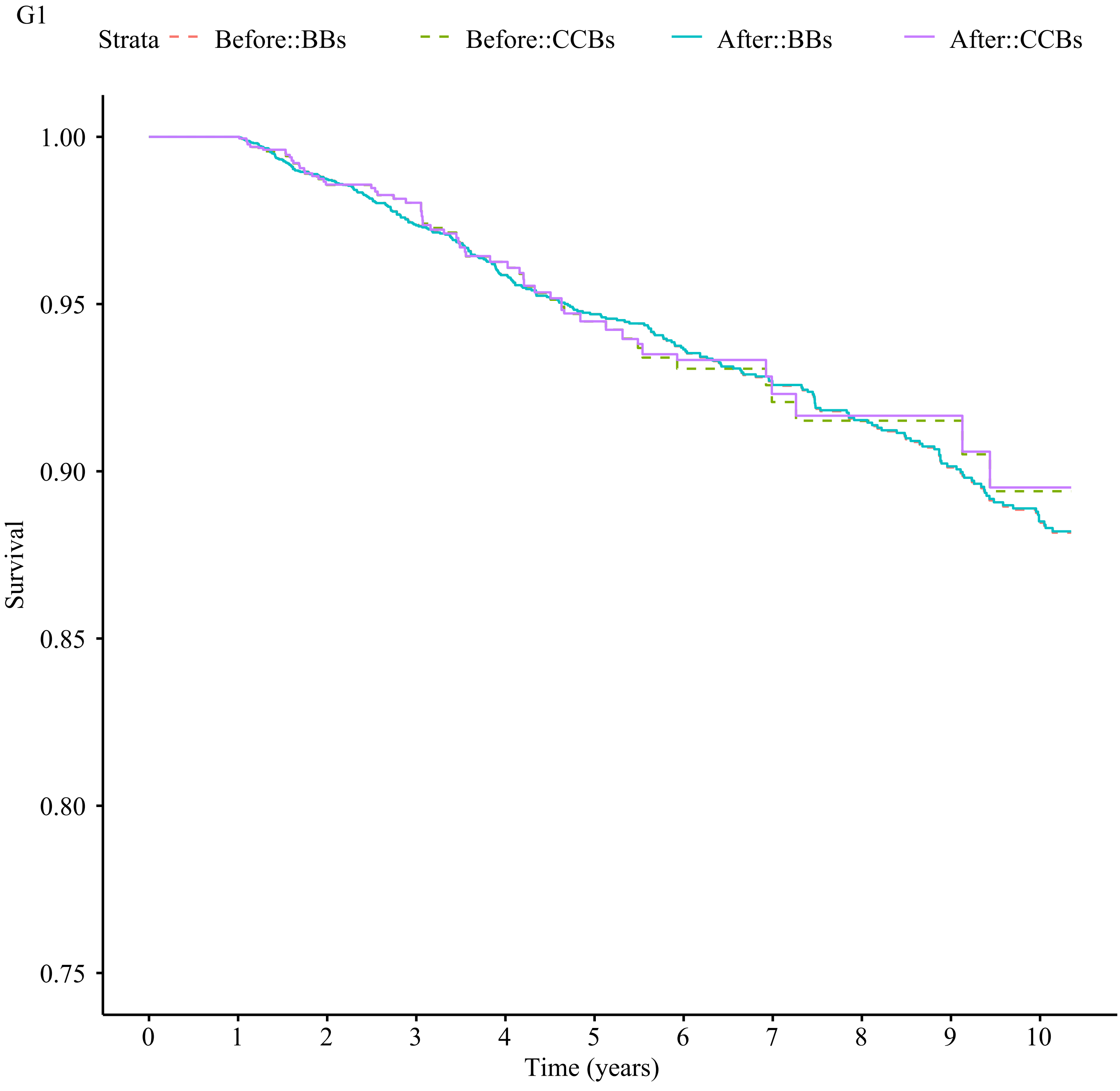
G1
Strata
Before::BBs
Before::CCBs
After::BBs
After::CCBs
1.00
0.95
0.90
0.85
0.80
0.75
Survival
0
1
2
3
4
5
6
7
8
9
10
Time (years)

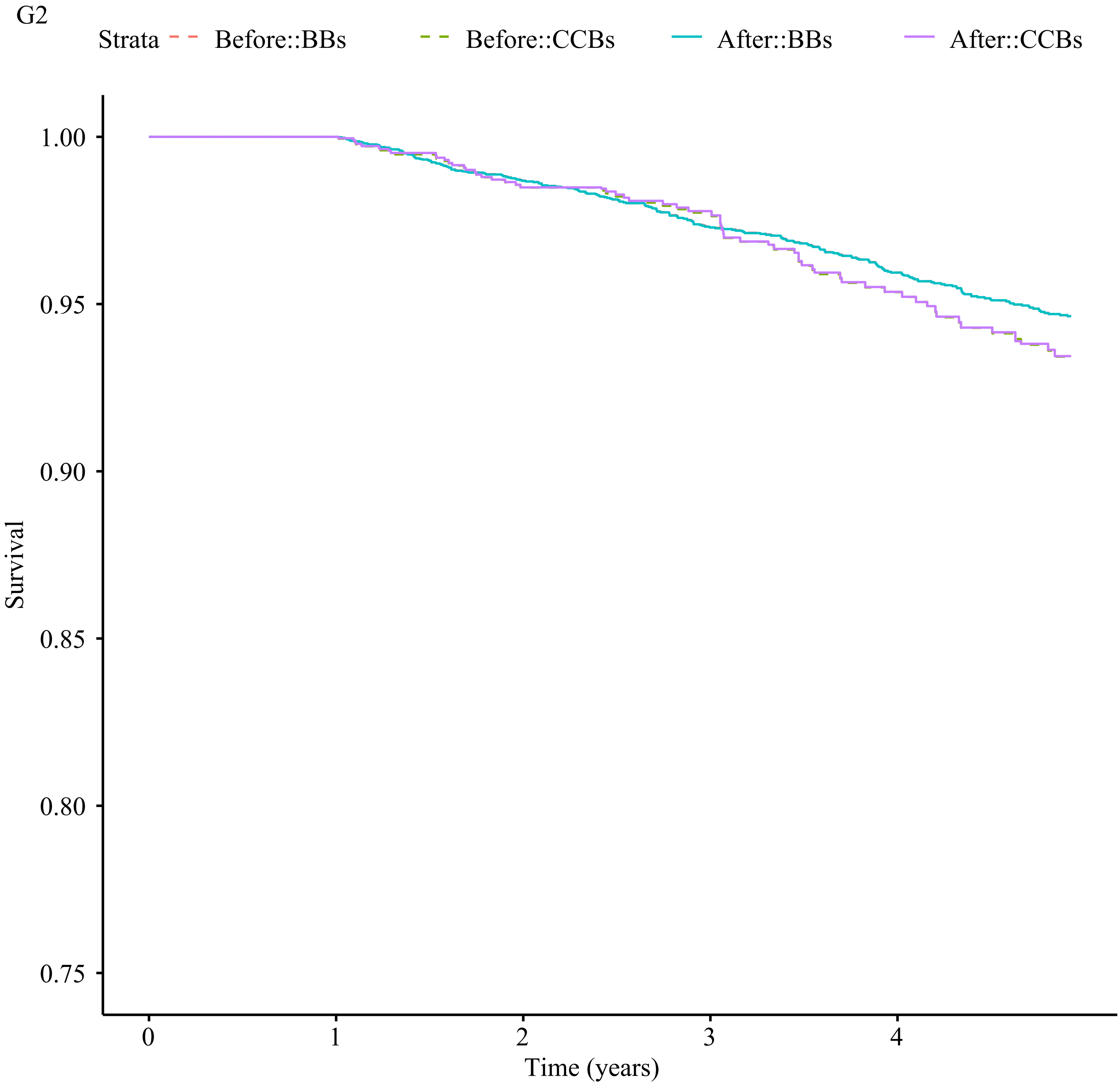

G2
Strata
Before::BBs
Before::CCBs
After::BBs
After::CCBs
1.00
0.95
0.90
0.85
0.80
0.75
Survival
0
1
2
3
4
Time (years)

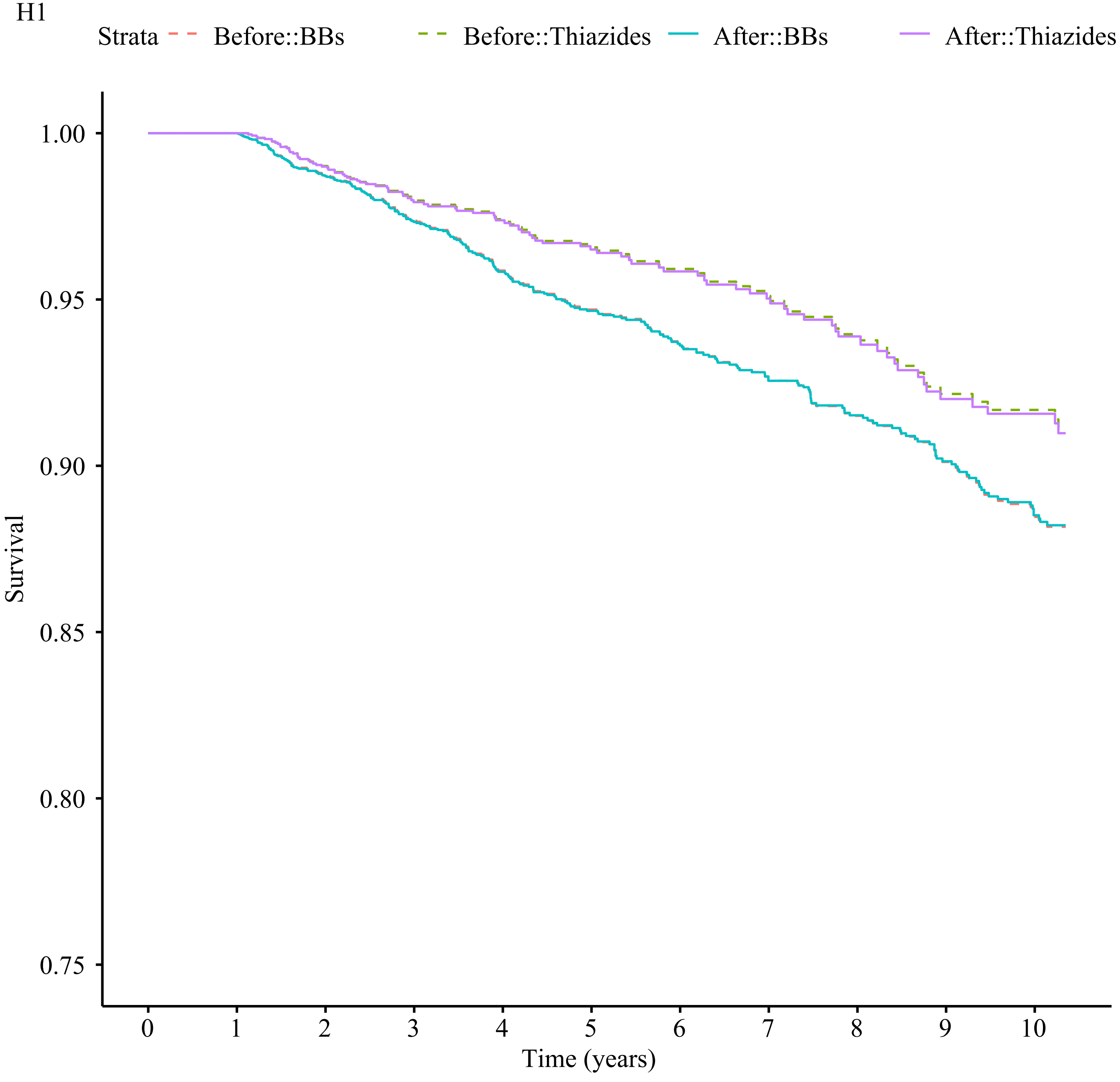
H1
Strata
Before::BBs
Before::Thiazides
After::BBs
After::Thiazides
1.00
0.95
0.90
0.85
0.80
0.75
Survival
0
1
2
3
4
5
6
7
8
9
10
Time (years)

H2

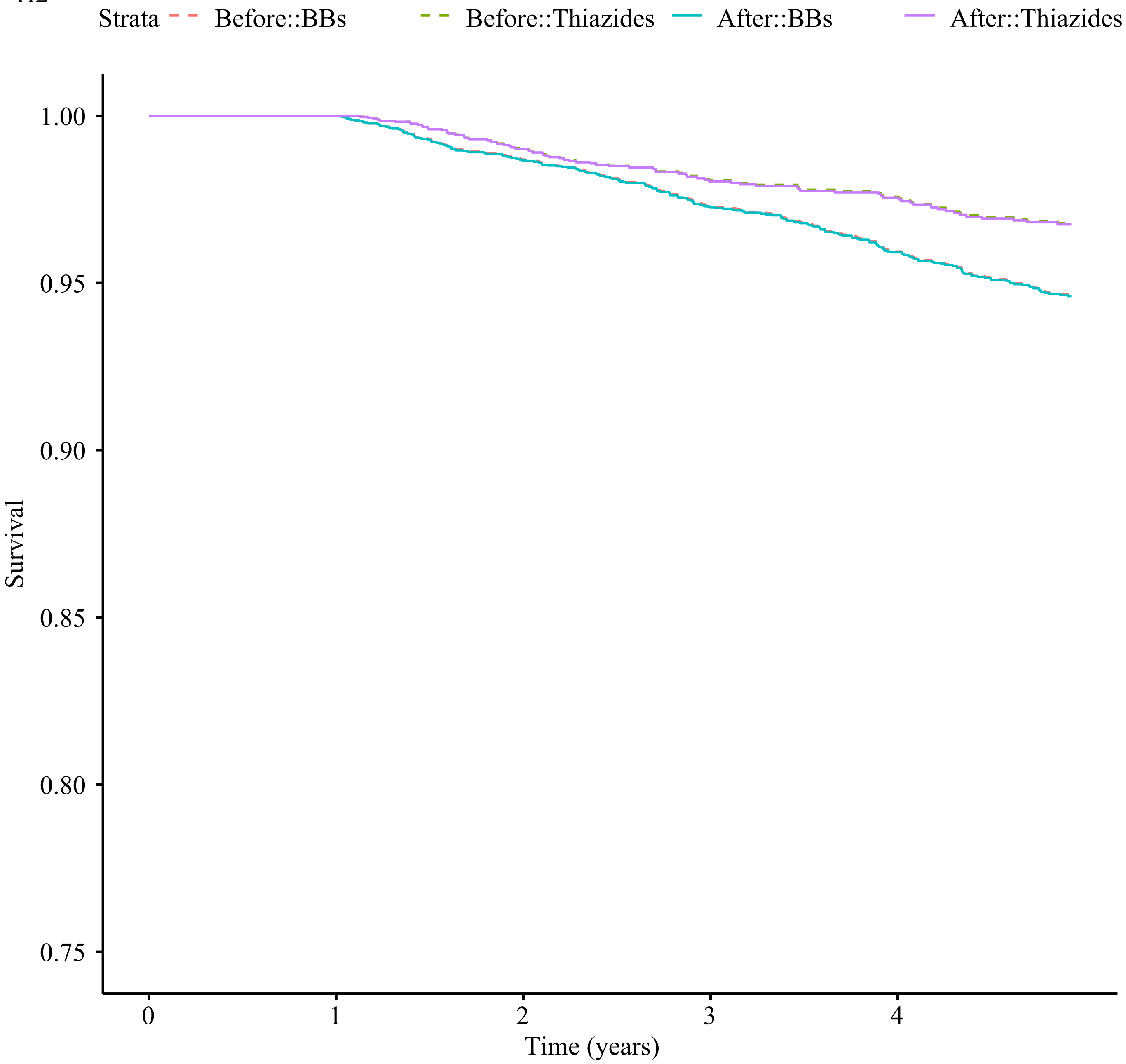

Strata
Before::BBs
Before::Thiazides
After::BBs
After::Thiazides
1.00
0.95
0.90
0.85
0.80
0.75
Survival
0
1
2
3
4
Time (years)

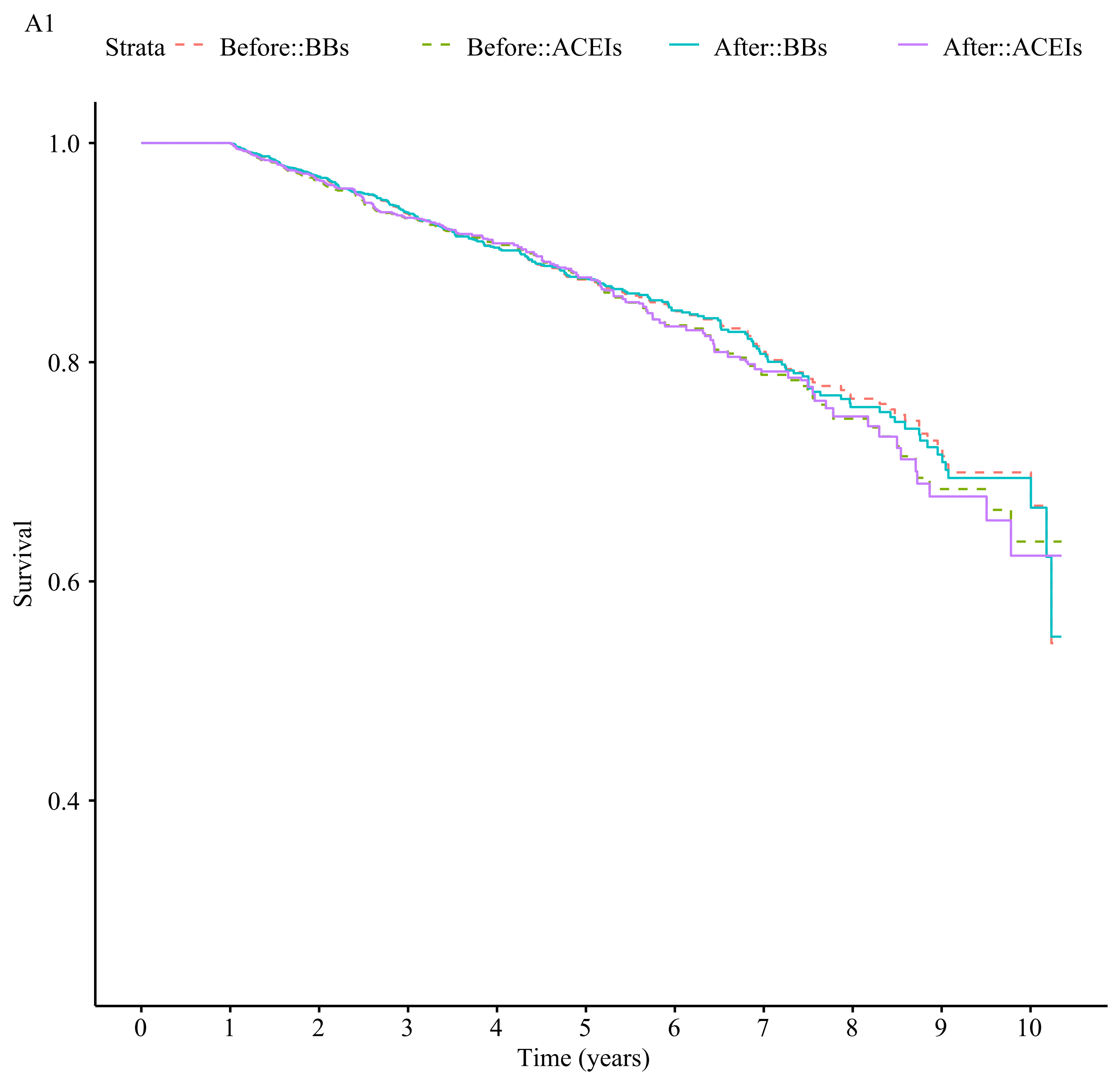

A1
Strata
Before::BBs
Before::ACEIs
After::BBs
After::ACEIs
1.0
0.8
0.6
0.4
Survival
0
1
2
3
4
5
6
7
8
9
10
Time (years)

A2

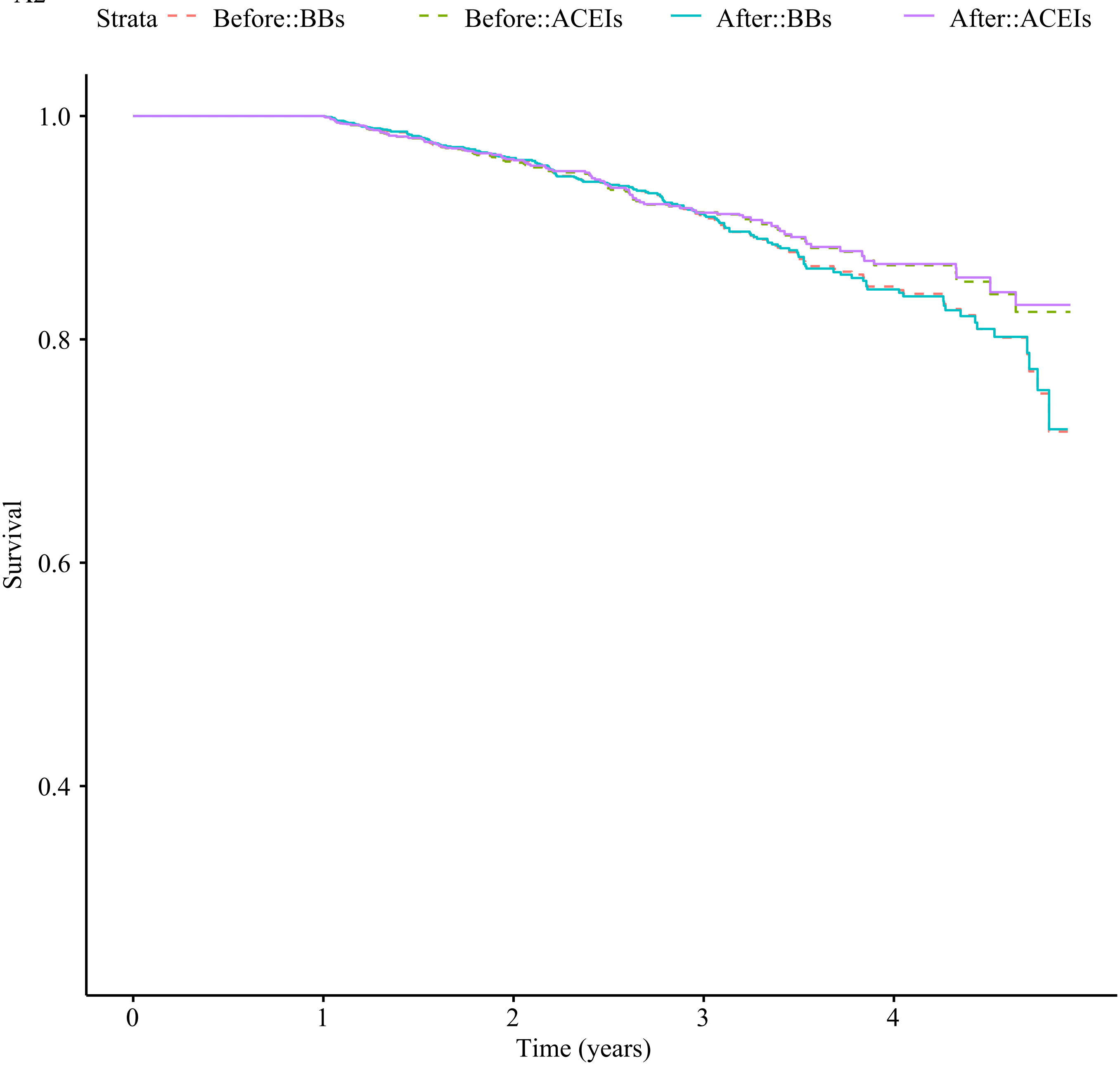
Strata
Before::BBs
Before::ACEIs
After::BBs
After::ACEIs
1.0
0.8
0.6
0.4
Survival
0
1
2
3
4
Time (years)

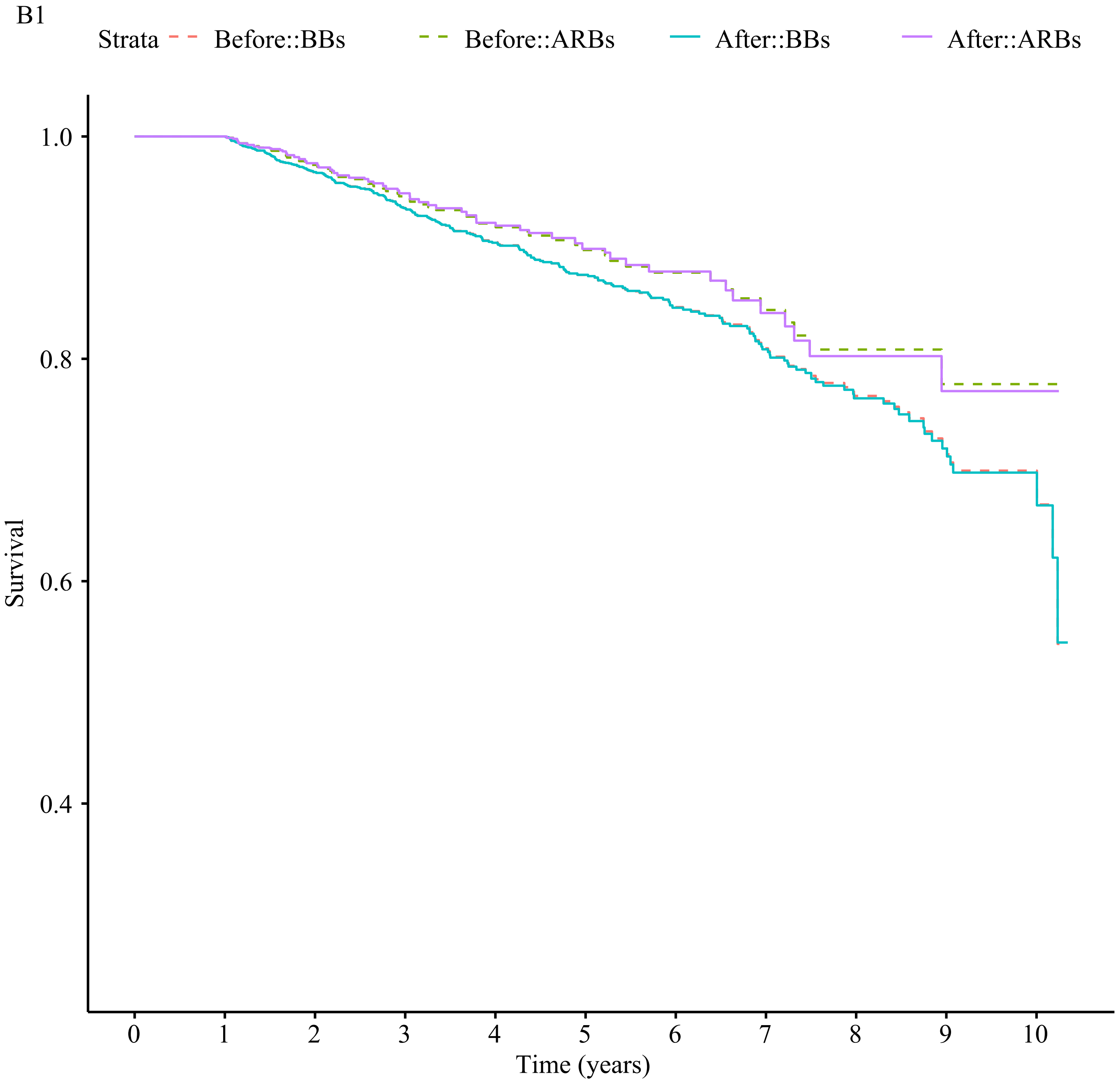
B1
Strata
Before::BBs
Before::ARBs
After::BBs
After::ARBs
1.0
0.8
0.6
0.4
Survival
0
1
2
3
4
5
6
7
8
9
10
Time (years)

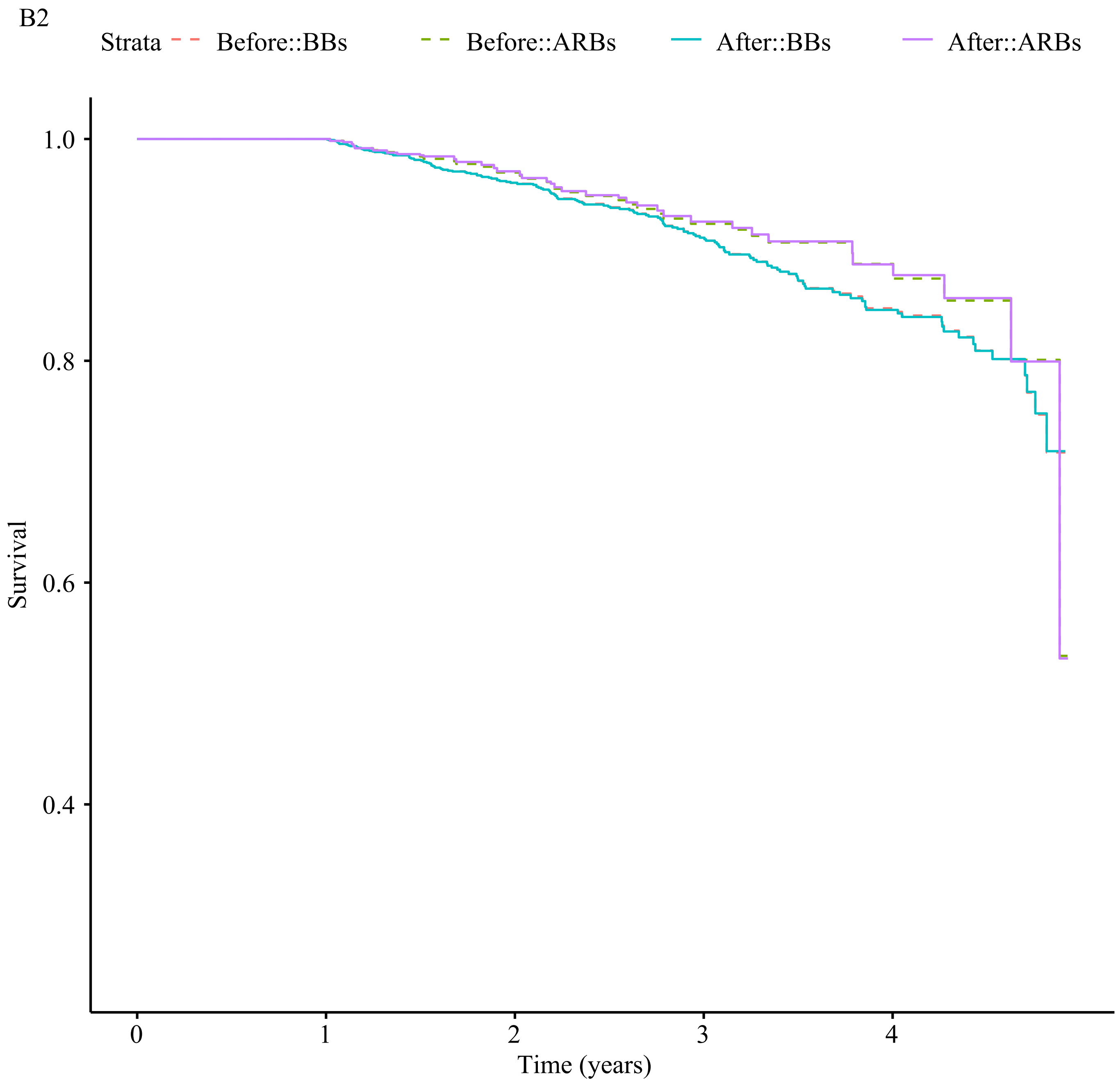
B2
Strata
Before::BBs
Before::ARBs
After::BBs
After::ARBs
Survival
1.0
0.8
0.6
0.4
0
1
2
3
4
Time (years)

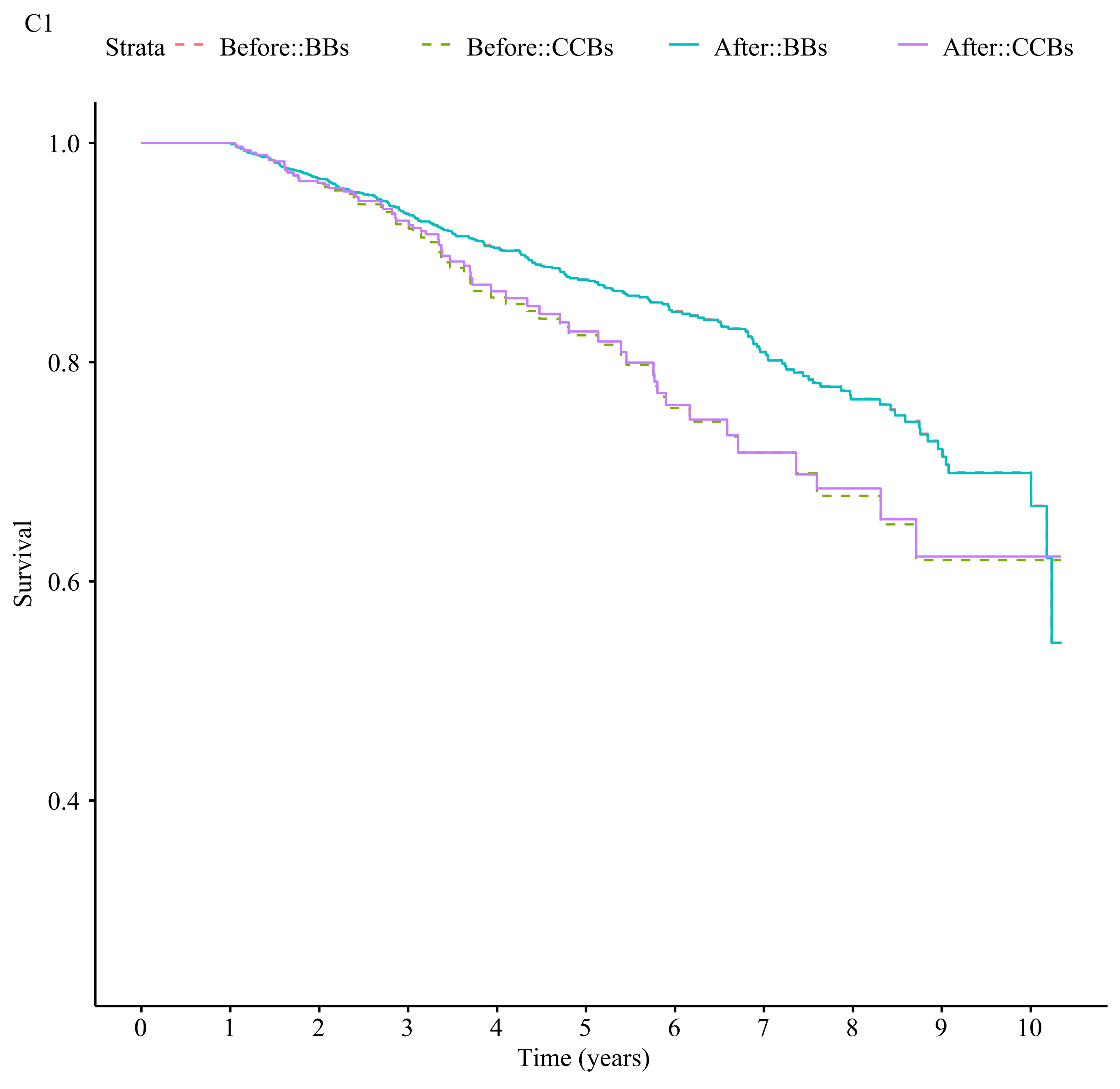
C1
Strata
Before::BBs
Before::CCBs
After::BBs
After::CCBs
Survival
1.0
0.8
0.6
0.4
0
1
2
3
4
5
6
7
8
9
10
Time (years)

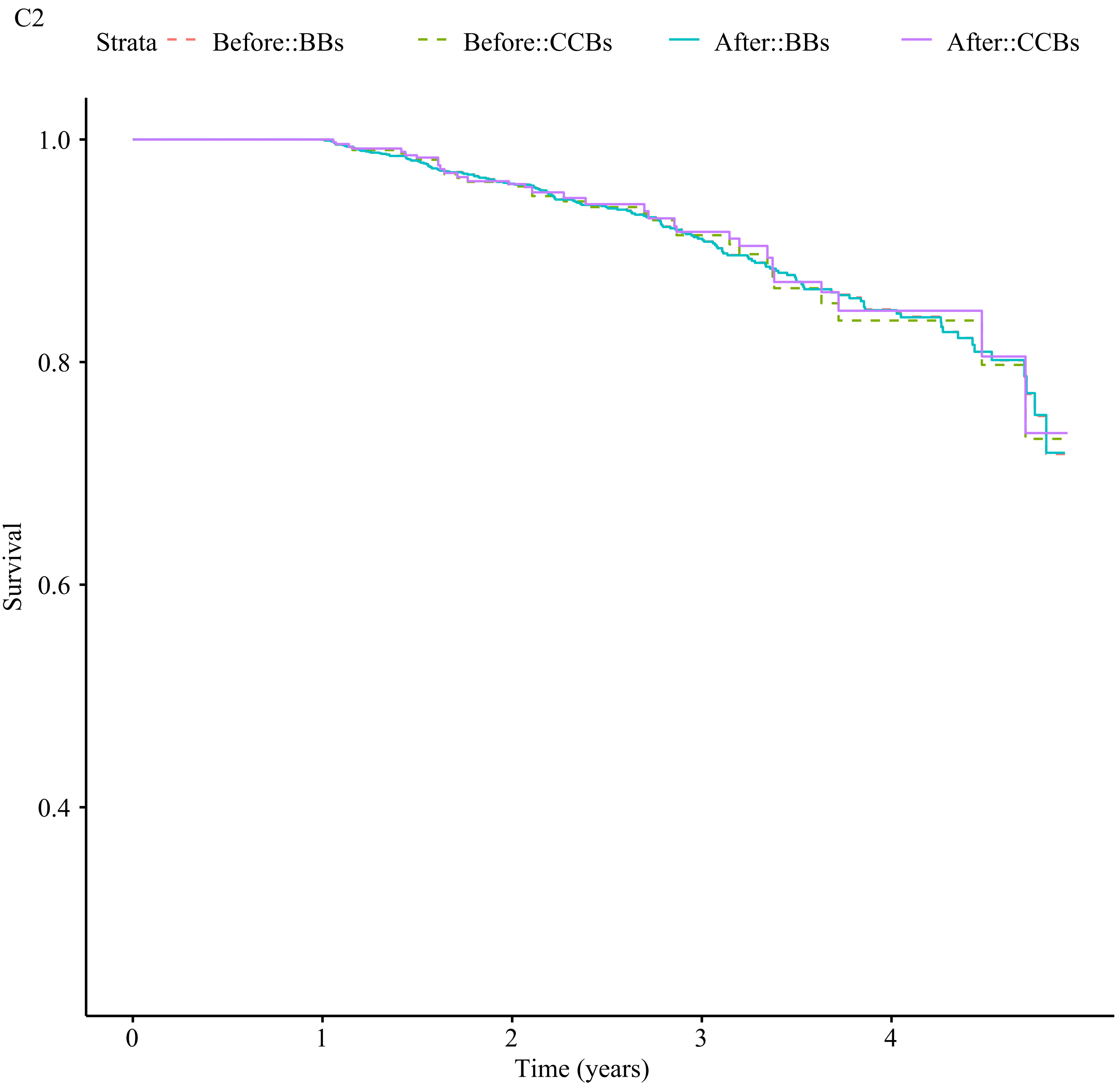
C2
Strata
Before::BBs
Before::CCBs
After::BBs
After::CCBs
Survival
1.0
0.8
0.6
0.4
0
1
2
3
4
Time (years)

D1

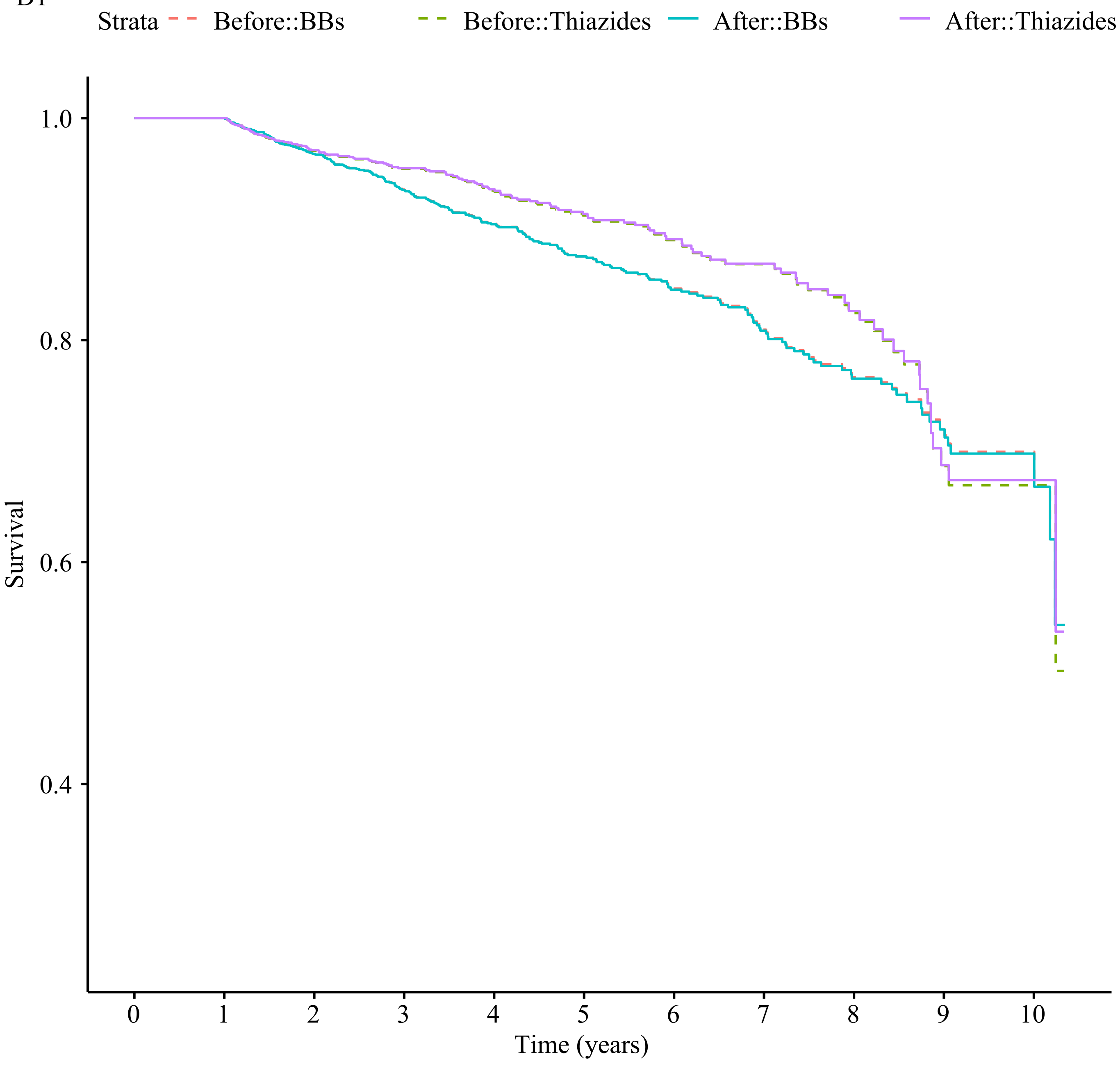

Strata
Before::BBs
Before::Thiazides
After::BBs
After::Thiazides
1.0
0.8
0.6
0.4
Survival
0
1
2
3
4
5
6
7
8
9
10
Time (years)

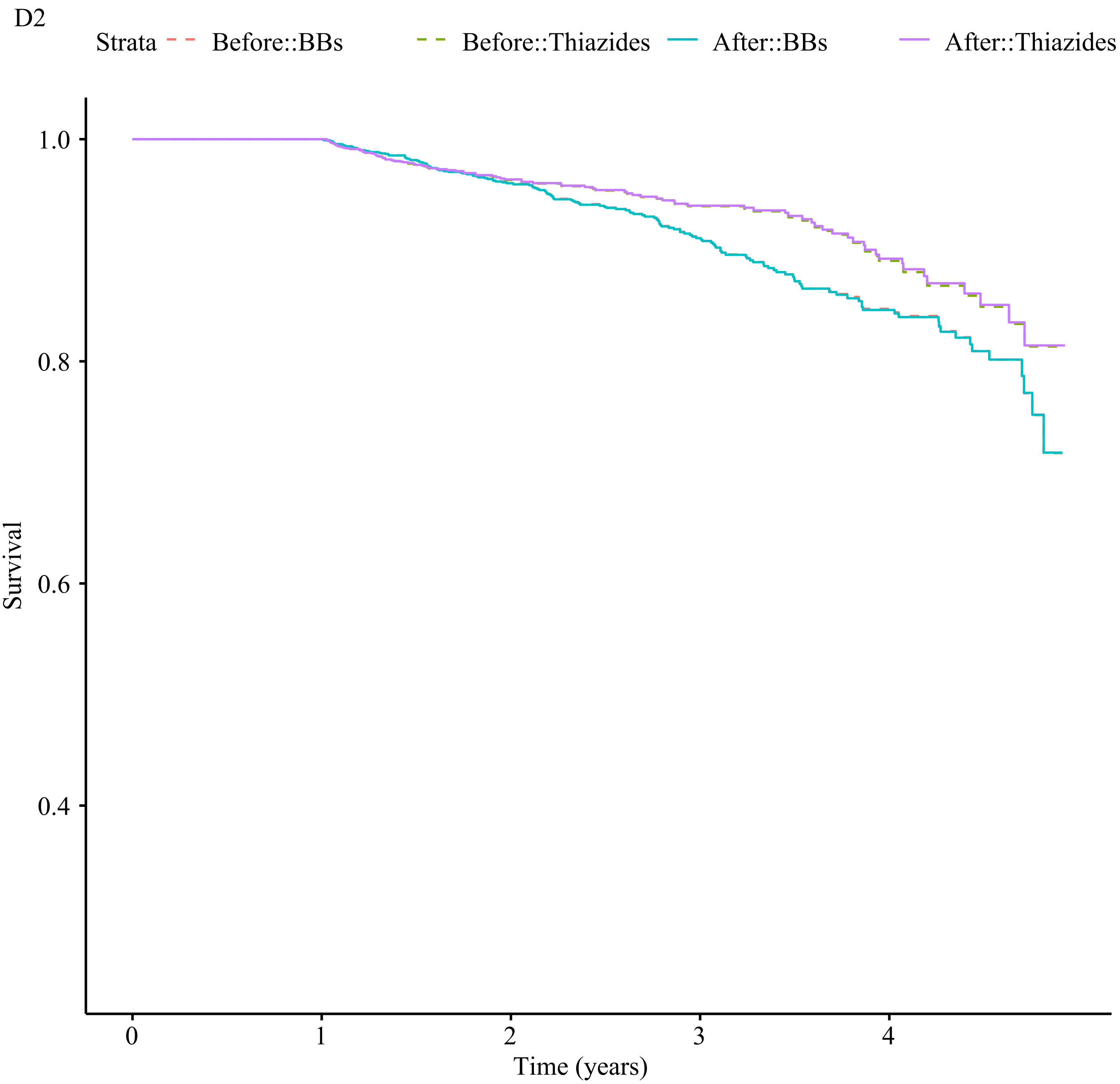
D2
Strata
Before::BBs
Before::Thiazides
After::BBs
After::Thiazides
Survival
1.0
0.8
0.6
0.4
Time (years)
0
1
2
3
4

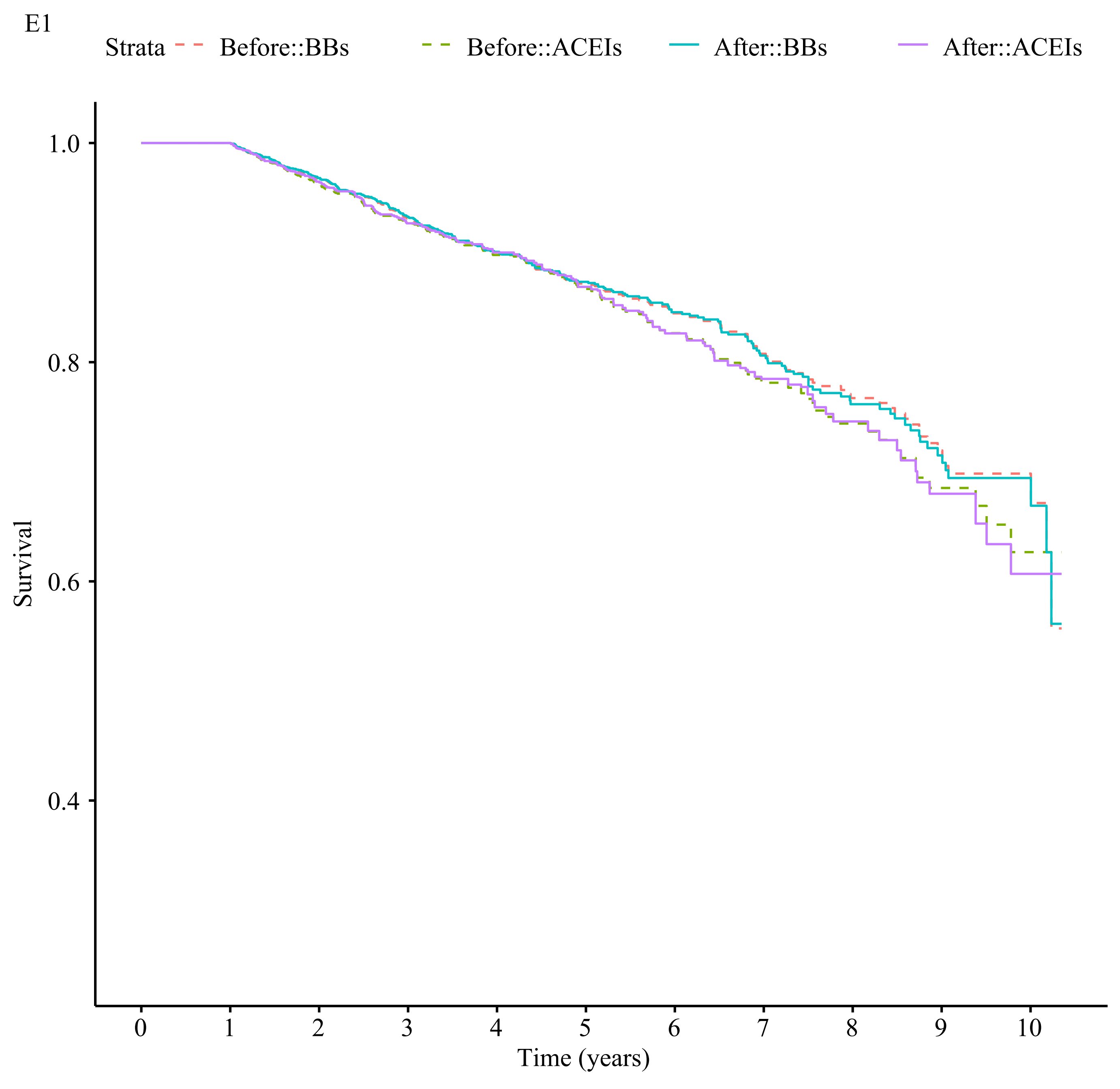

E1
Strata
Before::BBs
Before::ACEIs
After::BBs
After::ACEIs
1.0
0.8
0.6
0.4
Survival
0
1
2
3
4
5
6
7
8
9
10
Time (years)

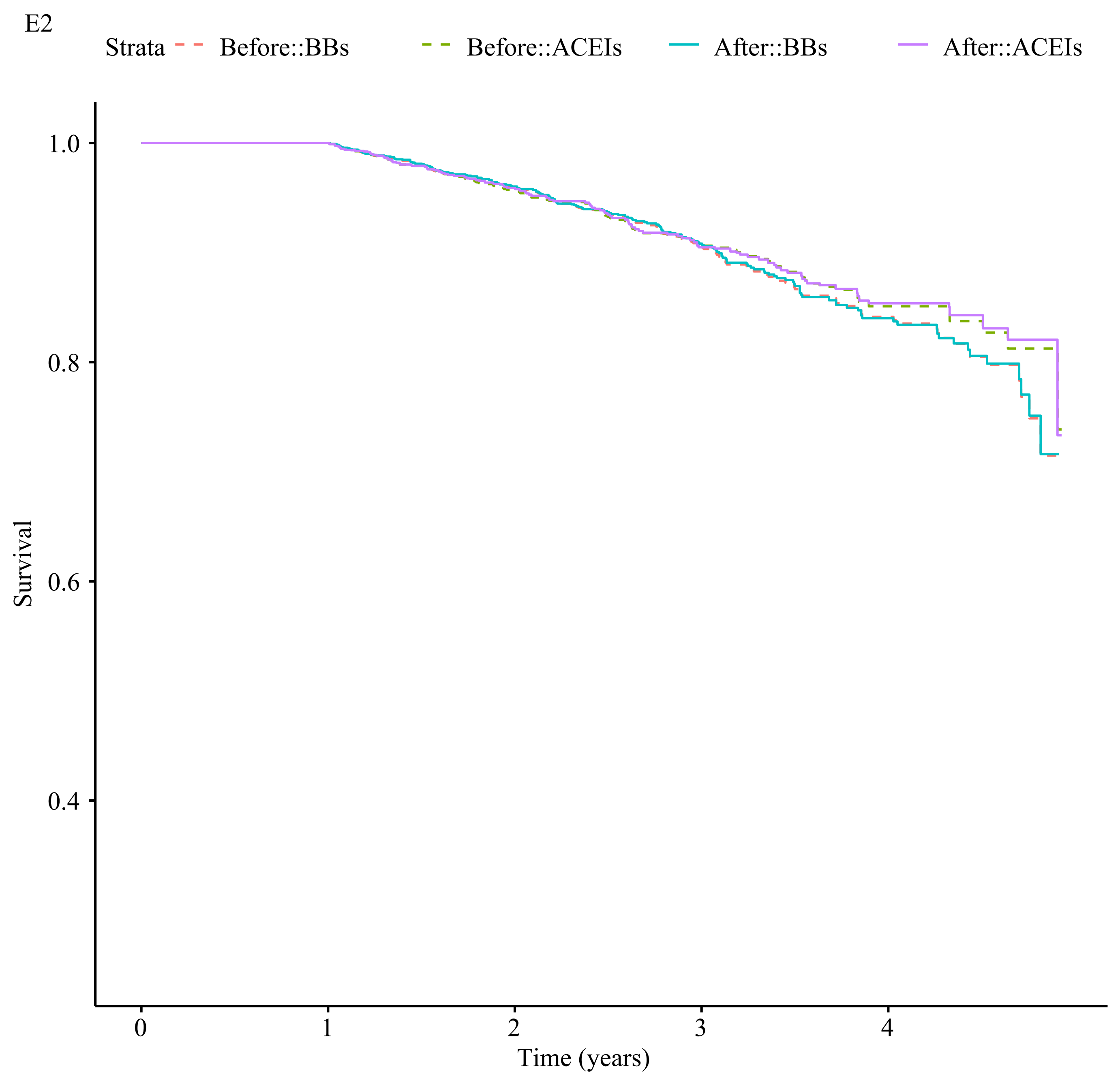

E2
Strata
Before::BBs
Before::ACEIs
After::BBs
After::ACEIs
1.0
0.8
0.6
0.4
Survival
0
1
2
3
4
Time (years)

F1

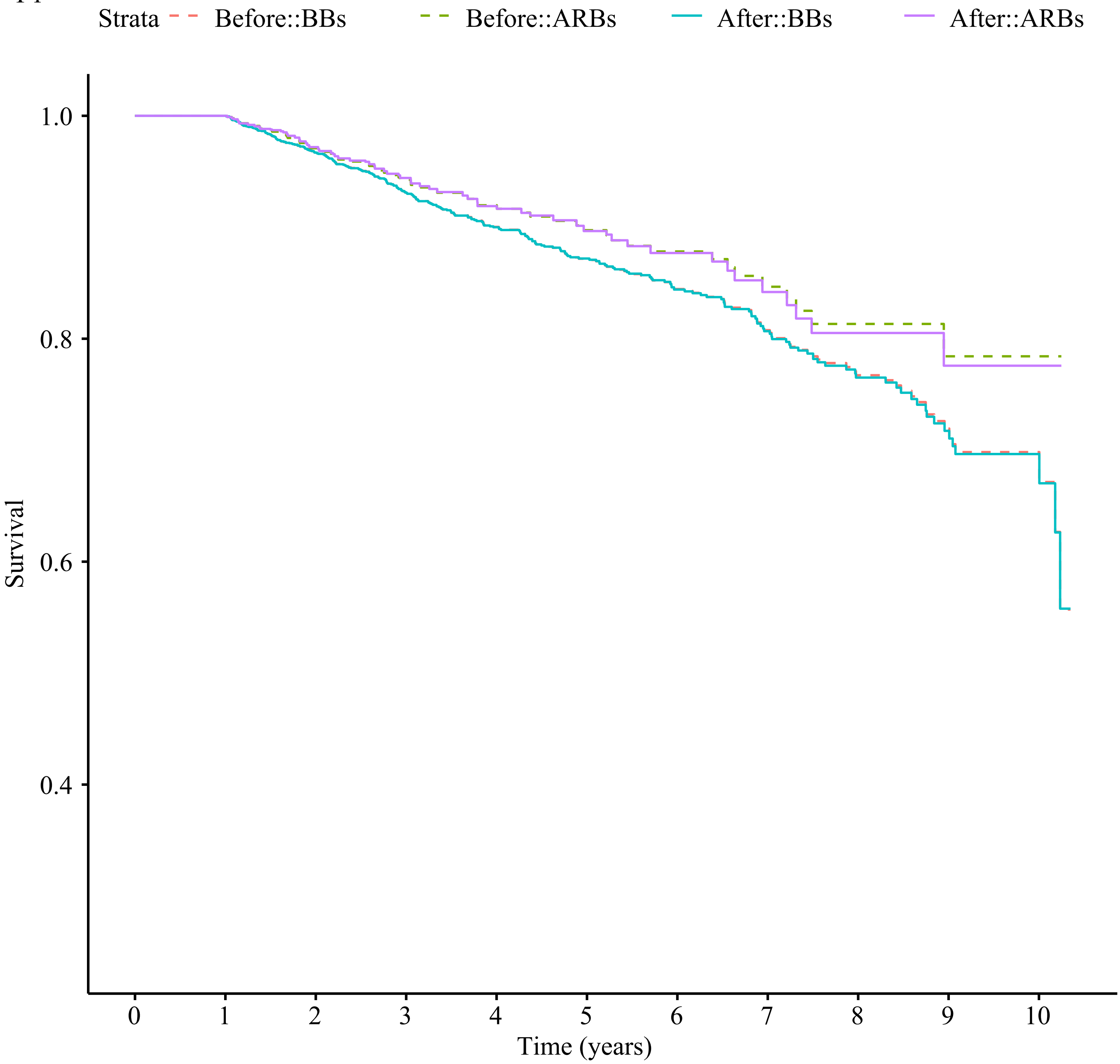

Strata
Before::BBs
Before::ARBs
After::BBs
After::ARBs
1.0
0.8
0.6
0.4
Survival
0
1
2
3
4
5
6
7
8
9
10
Time (years)

F2

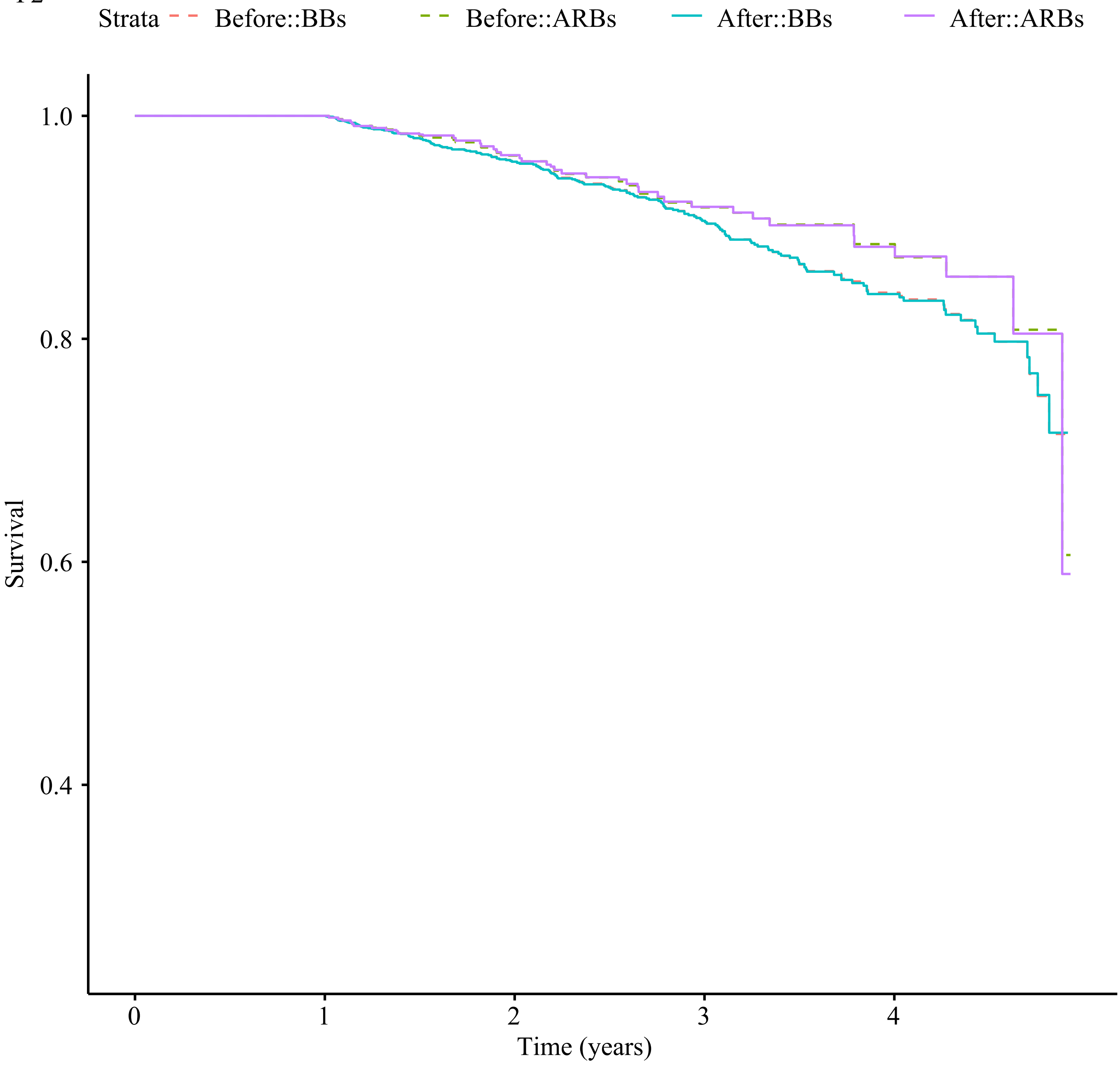

Strata
Before::BBs
Before::ARBs
After::BBs
After::ARBs
1.0
0.8
0.6
0.4
Survival
0
1
2
3
4
Time (years)

G1

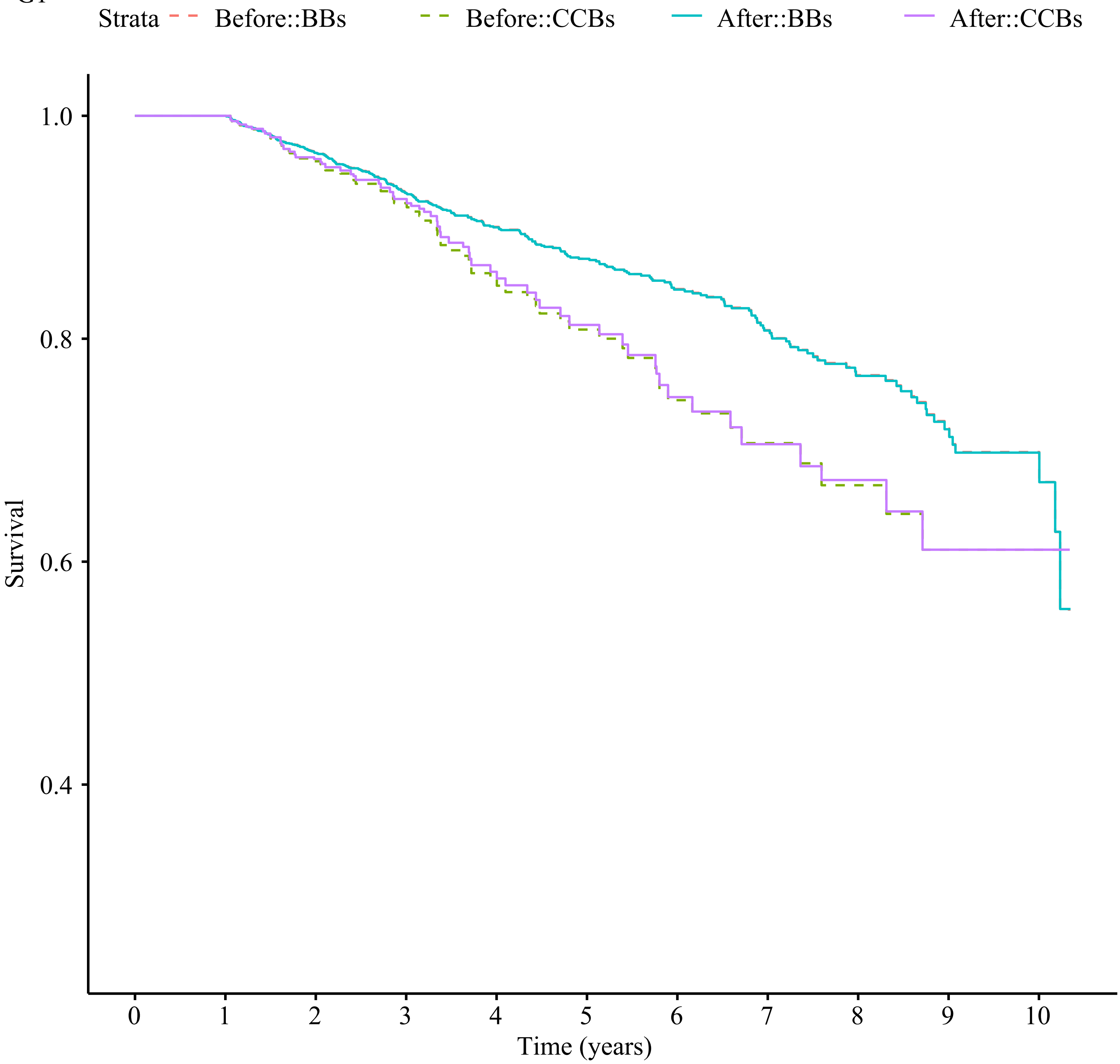
Strata
Before::BBs
Before::CCBs
After::BBs
After::CCBs
Survival
1.0
0.8
0.6
0.4
0
1
2
3
4
5
6
7
8
9
10
Time (years)

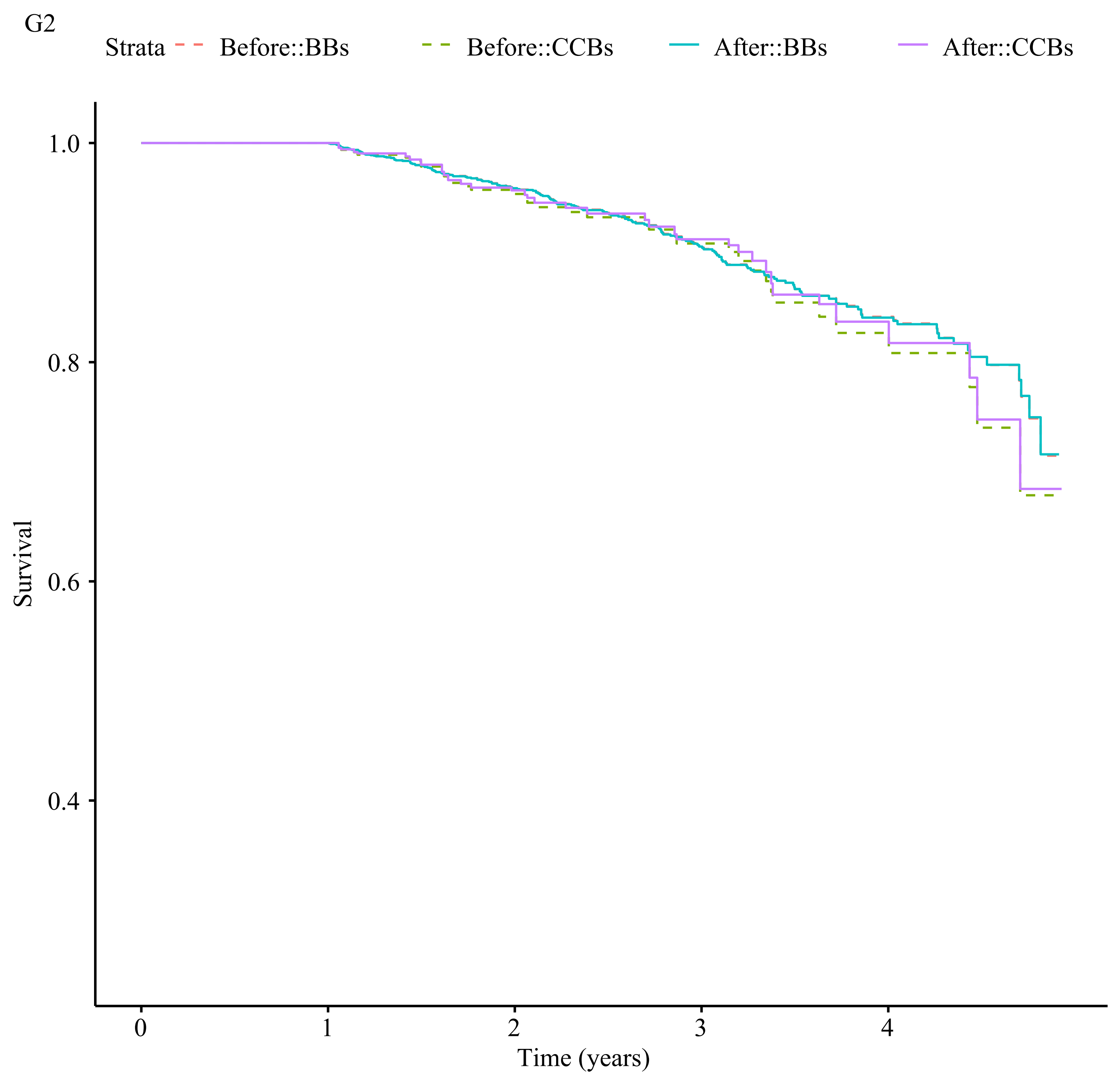

G2
Strata
Before::BBs
Before::CCBs
After::BBs
After::CCBs
1.0
0.8
0.6
0.4
Survival
0
1
2
3
4
Time (years)

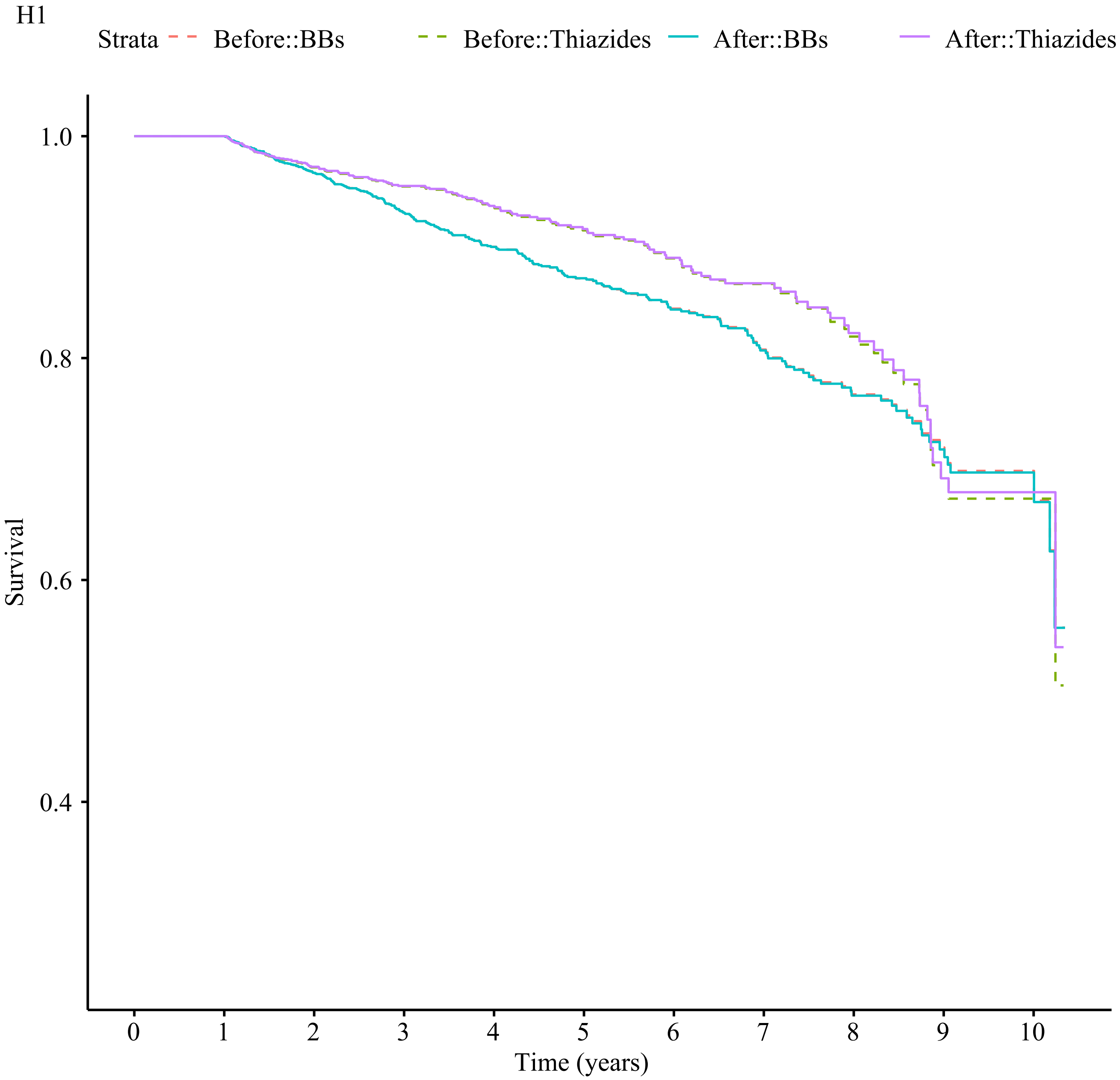

H1
Strata
Before::BBs
Before::Thiazides
After::BBs
After::Thiazides
1.0
0.8
0.6
0.4
Survival
0
1
2
3
4
5
6
7
8
9
10
Time (years)

H2

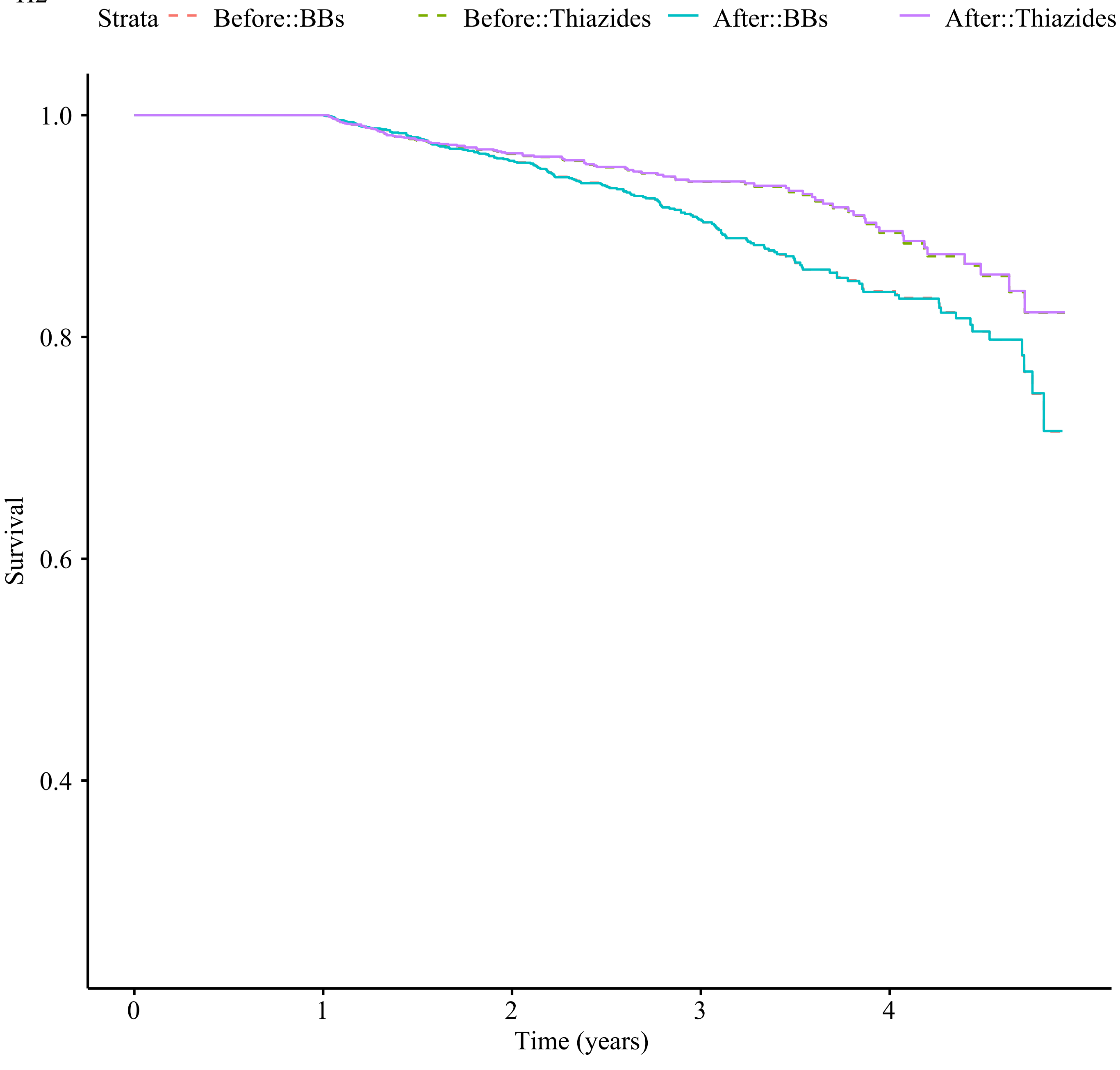

Strata
Before::BBs
Before::Thiazides
After::BBs
After::Thiazides
Survival
1.0
0.8
0.6
0.4
Time (years)
0
1
2
3
4
